# Membrane Chemistry Tunes the Structure of a Peptide Transporter†

**DOI:** 10.1002/anie.202008226

**Published:** 2020-09-11

**Authors:** Tanya Lasitza‐Male, Kim Bartels, Jakub Jungwirth, Felix Wiggers, Gabriel Rosenblum, Hagen Hofmann, Christian Löw

**Affiliations:** ^1^ Department of Structural Biology Weizmann Institute of Science Herzl St. 234 7610001 Rehovot Israel; ^2^ Department of Chemical and Biological Physics Weizmann Institute of Science Herzl St. 234 7610001 Rehovot Israel; ^3^ Centre for Structural Systems Biology (CSSB) DESY and European Molecular Biology Laboratory Hamburg Notkestrasse 85 22607 Hamburg Germany; ^4^ Department of Medical Biochemistry and Biophysics Karolinska Institutet 17177 Stockholm Sweden

**Keywords:** FRET, biomembranes, membrane proteins, protein structures, single-molecule studies

## Abstract

Membrane proteins require lipid bilayers for function. While lipid compositions reach enormous complexities, high‐resolution structures are usually obtained in artificial detergents. To understand whether and how lipids guide membrane protein function, we use single‐molecule FRET to probe the dynamics of DtpA, a member of the proton‐coupled oligopeptide transporter (POT) family, in various lipid environments. We show that detergents trap DtpA in a dynamic ensemble with cytoplasmic opening. Only reconstitutions in more native environments restore cooperativity, allowing an opening to the extracellular side and a sampling of all relevant states. Bilayer compositions tune the abundance of these states. A novel state with an extreme cytoplasmic opening is accessible in bilayers with anionic head groups. Hence, chemical diversity of membranes translates into structural diversity, with the current POT structures only sampling a portion of the full structural space.

## Graphical abstract

Single‐molecule FRET experiments show that the structure of the peptide membrane transporter DtpA is tuned by membrane composition. Head‐group chemistry determines which of three alternative conformations dominates. An extreme cytoplasmic opening is observed with anionic head groups.

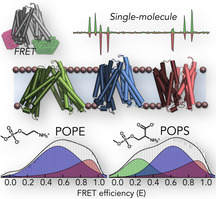

## Introduction

Membrane proteins with their ability to shuttle, pump, exchange, and transmit signals across lipid bilayers^1^, ^2^, ^3^, ^4^, ^5^, ^6^, ^7^ represent 30 % of proteins in living organisms^8^, ^9^ and 60 % of current drug targets.^10^, ^11^ While membranes are key for their function,^12^, ^13^, ^14^, ^15^, ^16^, ^17^ methods such as *x*‐ray crystallography or cryo‐electron microscopy often require detergent extraction for obtaining high‐resolution structures.^18^, ^19^, ^20^, ^21^, ^22^, ^23^, ^24^ Unfortunately, it is unclear whether structure and function of these pharmacologically important proteins are preserved in such artificial environments.^25^, ^26^ We address this question by comparing the conformations of a major facilitator superfamily (MFS) transporter between detergent and membrane‐mimicking environments using single‐molecule Förster resonance energy transfer (smFRET).

**Figure 1:**
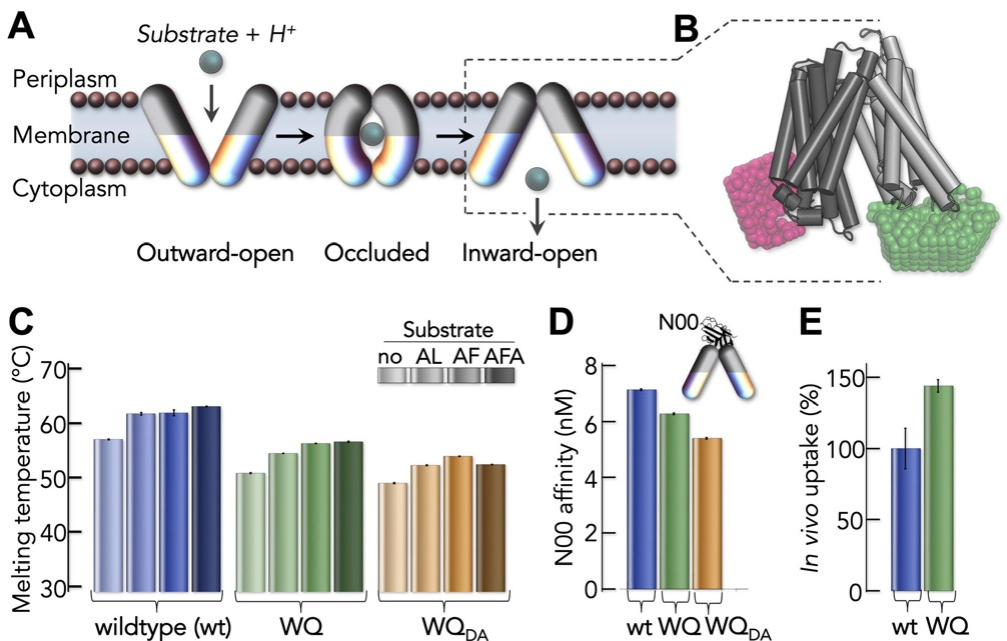
Transport mechanism and functionality of DtpA. A) Schematics of the alternate access mechanism. Substrate (gray sphere) transport requires alternate access of the substrate‐binding center to each membrane side. B) Structure of DtpA (gray), including the positional distribution (see Methods in the Supporting Information) of the FRET dyes (pink, green). C) Thermal stability of wildtype DtpA and the variant WQ in its unlabeled (WQ) and labeled (WQ_DA_) form in the absence and presence of different ligands (indicated). D) Nanobody affinity to wildtype DtpA (blue) and the variants WQ before (green) and after labeling (orange). E) In vivo AMCA uptake efficiency of wildtype DtpA and the variant WQ.

The MFS is one of the largest families of membrane transporters in nature.^27^, ^28^ It mediates the uptake of a broad spectrum of substrates and is therefore key for maintaining cell homeostasis.^29^ MFS transporters work via an alternate access mechanism^30^, ^31^ that requires at least three states:^32^, ^33^ inward‐open, occluded, and outward‐open (Figure 1 A). Members of the MFS share a common fold:^34^ twelve transmembrane helices organized in two six‐helix bundles (Figure 1 B). A particularly important subfamily are proton‐dependent oligopeptide transporters (POTs)^27^ that utilize a proton gradient^35^ to uptake di‐ and tripeptides but also peptide‐mimetic drugs.^36^, ^37^ Their remarkable substrate promiscuity^38^, ^39^ makes them important targets for drug delivery. Numerous bacterial POT structures have been determined to date.^40^, ^41^, ^42^, ^43^, ^44^, ^45^, ^46^, ^47^, ^48^, ^49^, ^50^, ^51^, ^52^, ^53^, ^54^, ^55^, ^56^, ^57^ We focus on the *E. coli* peptide transporter DtpA^58^, ^59^ (Figure 1 B), a homolog of human PepT1 and PepT2.^36^, ^60^, ^61^, ^62^, ^63^, ^64^, ^65^, ^66^ Its similar substrate specificity compared to human homologs makes DtpA an ideal prototype to understand the mechanism of peptide and drug transport.^48^ However, current *x*‐ray structures of POTs in detergents revealed mainly inward‐open conformations,^40^, ^43^, ^44^, ^45^, ^46^, ^47^, ^48^, ^49^, ^51^, ^52^ thus hampering an atomic level understanding of the transport cycle. This bias may be symptomatic for non‐natural detergent environments, thus calling for in‐depth comparison of POT conformations in detergent and lipid environments.

## Results and Discussion

We labeled DtpA on its cytoplasmic side (Figure 1 A,B and SI Appendix, Figure S1) using the variant W203C/Q487C (WQ). The intrinsic cysteines were replaced by serine residues. WQ is fully functional in binding ligands (Figure 1 C and SI Appendix, Figure S2–5) and a conformation‐specific nanobody (N00) (Figure 1 D and SI Appendix, Figure S6,7, Table S2), and it shows high transport activity in an in vivo uptake assay (Figure 1 E and SI Appendix, Figure S8). Similarly, labeling with donor (Alexa Fluor 488) and acceptor (Alexa Fluor 594) neither impaired the thermal stability of the transporter (Figure 1 C and SI Appendix, Figure S3), nor N00 binding (Figure 1 D and SI Appendix, Figure S6,7, Table S2). Notably, an alternative variant W203C/T351C (WT) shows results identical to those of the WQ variant (SI Appendix, Figure S1–3, S7–9, S10–12, Table S2).

### Structure and Dynamics of DtpA in Detergent

**Figure 2:**
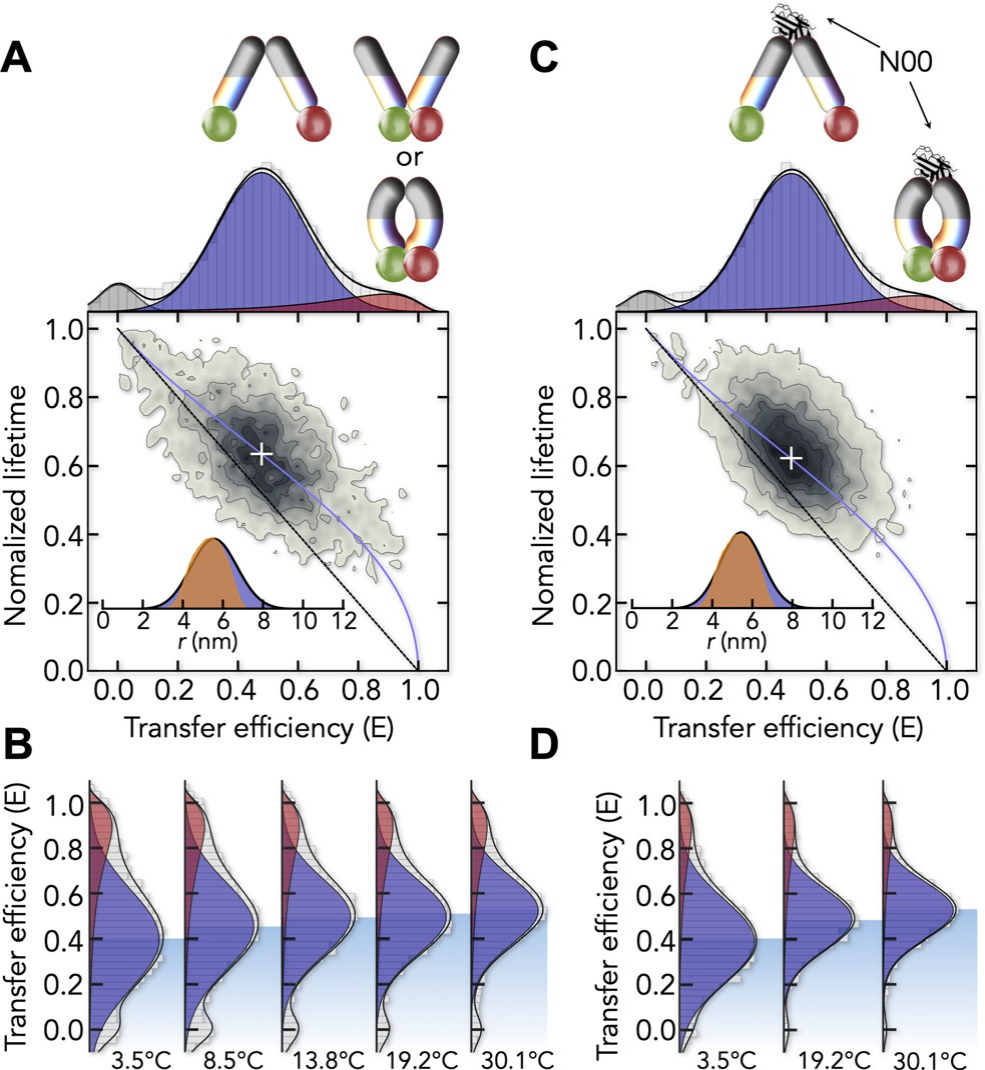
SmFRET of DtpA in detergent. A) FRET histogram of WQ_DA_ in LMNG (top). Solid lines are fits with Gaussian and log‐normal peaks to account for open (blue) and closed (red) molecules. Molecules that lack an active acceptor are shown in gray. (bottom) 2D correlation map between donor fluorescence lifetime and transfer efficiency. Solid lines show the dependence for a single donor‐acceptor distance (black) and the best fit of the mean position of open molecules (white cross) with a distance distribution (blue). Inset: The distance distribution obtained from the fit (blue) and the expected distance distribution for the rigid X‐ray structure assuming a static positional distribution of the dyes (orange). B) FRET histograms of DtpA at different temperatures. The position of the open population is indicated by the shaded area. C,D) Same as (A,B) in the presence of N00 (8 μm).

The detergent LMNG (lauryl‐maltose‐neopentyl‐glycol) is commonly used to extract membrane proteins and to determine their structure using *x*‐ray crystallography and cryo‐EM.^20^, ^67^, ^68^, ^69^, ^70^, ^71^, ^72^, ^73^, ^74^, ^75^, ^76^, ^77^ We therefore probed the conformation of freely diffusing DtpA in LMNG. The FRET histogram shows two peaks: a minor peak at high FRET (*E*=0.89) and a major peak at lower FRET (*E*=0.48) (Figure 2 A). Although the FRET efficiency of the major peak agrees with the value computed from the *x*‐ray structure of the inward‐open state (0.54) (Methods), its donor fluorescence lifetime deviates from the expected value for a single donor‐acceptor distance (Figure 2 A), suggesting a distribution instead of a defined structure.^78^, ^79^, ^80^ A fit with an empirical model^81^ results in a distance distribution width of 1.1 nm (Methods), suggesting variability in the opening of the cytoplasmic side (Figure 2 A, inset). Notably, a static distribution of dye rotamers on a rigid *x*‐ray structure would only cause a distribution width of 0.78 nm (Figure 2 A, inset). Yet, since fluorescence anisotropy measurements show that the dyes experience sufficient rotational freedom (Figure S12) at the timescale of the donor fluorescence lifetime, the distribution of dye positions only contributes moderately to the donor lifetime. Importantly, the structural variability of DtpA is accompanied by substantial pliability. With decreasing temperature, the FRET efficiency of the major peak shifts to lower values, thus causing an even wider opening (Figure 2 B). The result is remarkable because positional shifts of FRET peaks indicate (i) reconfiguration timescales faster than the diffusion of the protein through the confocal spot of our microscope (<1 ms) and (ii) large‐scale structural adaptations to external conditions, which are known for intrinsically disordered and unfolded proteins,^82^, ^83^ but not for well‐folded proteins such as DtpA. Contrary to the picture sketched by the *x*‐ray structure of DtpA, the results show that the inward‐open form of DtpA is a flexible conformational ensemble that is sampled at sub‐millisecond timescales. However, what is the structural origin of the minor high‐FRET population?

Given our labeling at the cytoplasmic side (Figure 1 B), molecules with high FRET exhibit a closed cytoplasmic side. Based on the alternate access model,^30^, ^31^, ^33^ a closed cytoplasmic side is indeed expected for outward‐open or occluded conformations (Figure 1 A). Unfortunately, the sensitivity of our FRET‐probes (2–8 nm) is insufficient to distinguish between these states and we therefore used a biochemical approach. The addition of the conformation‐specific nanobody (N00) blocks the opening of the periplasmic side (Figure 2 C). If the high‐FRET peak represents outward‐open molecules, blocking the periplasmic opening is expected to shift the equilibrium towards the low‐FRET peak (inward‐open) due to the mutually exclusive nature of these states.

However, despite a nanomolar affinity for DtpA (Figure 1 D, Table S2), N00 neither changes the abundance of the high‐FRET peak (Figure 2 C) nor does it alter the temperature adaptation of the inward‐open ensemble (Figure 2 D). Two scenarios could explain this result: either the high‐FRET peak corresponds to an occluded conformation with simultaneously closed cytoplasmic and periplasmic sides, or the high‐FRET peak is not in equilibrium with the open ensemble, suggesting that it represents kinetically trapped misfolded species. To exclude the latter, we checked whether the high‐ and low‐FRET peaks are in dynamic exchange.

**Figure 3:**
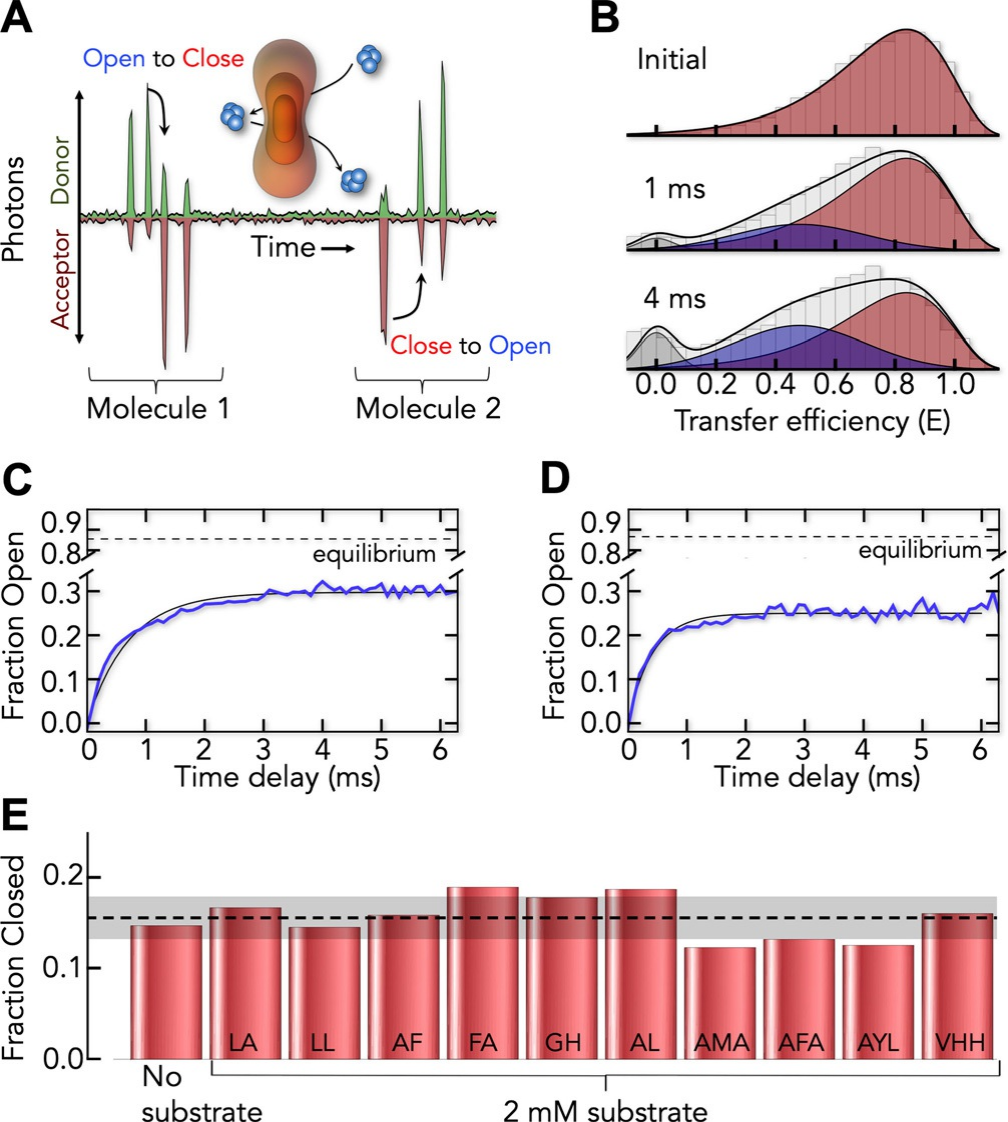
Dynamic exchange between high‐ and low‐FRET peaks. A) Schematics of a single‐molecule time trace of donor (green) and acceptor (red) signal. The multiple detection of two molecules due to their recurring passages through the confocal spot (orange) is shown. Switching between states is indicated (arrows). B) Single‐molecule recurrence histograms at two‐time delays (indicated) after initially selecting high‐FRET molecules (initial) show the time‐dependent rise of low‐FRET species. C) Time trace of the formation of low‐FRET molecules (blue) and a single‐exponential fit (black line). Dashed line indicates the equilibrium fraction of the low‐FRET population. D) Same as (C) in the presence of 8 μm N00. E) Relative abundance of high‐FRET molecules in the absence and presence of di‐ and tri‐peptides (indicated).

Here, we made use of the fact that a diffusing molecule may enter and exit the confocal spot numerous times (Figure 3 A). Once a molecule leaves the observation volume, the likelihood of it returning to this volume within a short time period is larger than the chance of detecting a new molecule. Hence, conformational switching events between successive transits can be identified (Figure 3 A), which allows us to probe dynamics in the regime of several milliseconds.^84^, ^85^ To extract the exchange kinetics, we binned the photon trace (100 μs steps) and selected all molecules in the high‐FRET population (0.7<*E*<1.2). We then constructed FRET histograms of those bins that followed the original set with a time delay. With increasing time delay, the high‐FRET population decreases and the low‐FRET population increases (Figure 3 B). The kinetics of forming low‐FRET molecules from the high‐FRET species follows single‐exponential kinetics with a relaxation time of ≈1 ms (Figure 3 C) that clearly demonstrates an exchange between the peaks. Misfolding can therefore be excluded as an origin of the high‐FRET peak. Notably, full equilibration is not reached within the accessible time window (Figure 3 C), indicating that also slower processes contribute to the exchange. Importantly, the exchange is also observed in the presence of N00 (Figure 3 D), which shows that opening and closing of the cytoplasmic side is independent of locking the periplasmic side. This loss in cooperativity between sides strongly indicates that the high‐FRET peak represents occluded molecules with simultaneously closed cytoplasmic and periplasmic sides. Indeed, such states have previously been found in detergent‐based *x*‐ray structures,^43^, ^46^, ^86^ but the timescale of their formation had been elusive.

Occluded molecules are intermediates on the path from outward‐open to inward‐open states (Figure 1 A). Given the underrepresentation of outward‐open *x*‐ray structures,^40^, ^41^, ^42^, ^43^, ^44^, ^45^, ^46^, ^47^, ^48^, ^49^, ^50^, ^51^, ^52^, ^53^, ^54^, ^55^, ^56^, ^57^ we utilized our smFRET approach to identify detergent conditions that stabilize the outward‐open conformation. To this end, we screened a broad spectrum of ligands (Figure 3 E and SI Appendix, Figure S13) with the goal to shift the balance between high‐ and low‐FRET peaks. However, none of the ligands and not even changes in the protonation state of DtpA (SI Appendix, Figure S14) altered the balance between high‐ and low‐FRET peaks significantly, suggesting that detergent does not provide the means to stabilize outward‐open molecules. This raises the general question of whether outward‐open states are sampled at all in the absence of proton‐ and substrate‐gradients. To address this question, we studied DtpA in membrane‐like nanoparticles stabilized by the protein Saposin A (SapNPs).^87^, ^88^

### Conformation and Dynamics of DtpA in SapNPs

**Figure 4:**
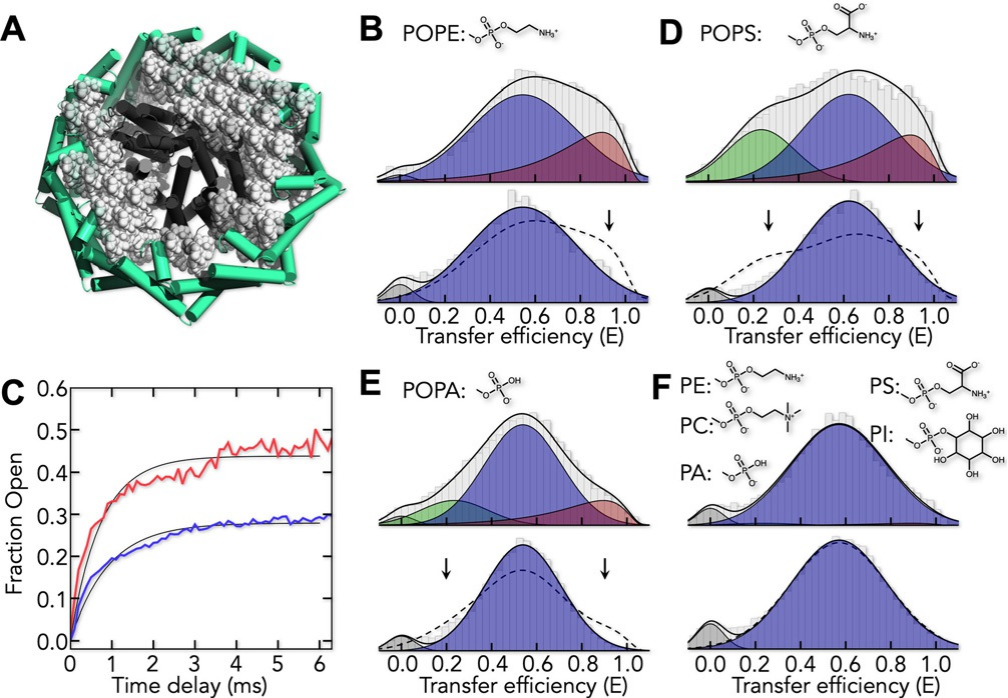
Conformational distribution of DtpA in SapNPs. A) Schematics of DtpA (dark gray) in a lipid SapNP stabilized by saposin A (green). Lipids are shown in gray. B) SmFRET histogram of DtpA in POPE SapNPs in the absence (top) and presence (bottom) of 8 μm N00. The position and width of the low‐FRET peak had first been determined in the presence of N00. The same parameters were then used to fit the histogram in the absence of N00. C) Time trace of the formation of low‐FRET molecules in POPE SapNPs in the absence (blue) and presence (red) of N00. Black line is a single‐exponential fit of the data. D–F) Same as B in SapNPs composed of POPS (D), POPA (E), and brain lipid extract (F). In eukaryotic brain lipid extract, 58.7 % of the lipid composition is unspecified by the manufacturer but likely contains >50 % PC.^90^

We reconstituted DtpA in SapNPs composed of POPE (1‐Palmitoyl‐2‐oleoyl‐sn‐glycero‐3‐phosphoethanol‐amine), the most abundant phospholipid in *E. coli* 89 (Figure 4 A and SI Appendix, Figure S4, S15,16). The FRET histogram of DtpA in SapNPs differs from that obtained in detergent (Figure 4 B).

While the peaks at low and high FRET efficiency are preserved, the histograms are broader. Still, the fluorescence lifetimes indicate that the peaks correspond to a distribution of conformers (SI Appendix, Figure S16). Since control experiments exclude quenching and a sticking of the FRET‐dyes to the protein surface (SI Appendix, Figure S12, S17), we conclude that peak broadening is due to a slower sampling within the open and closed ensembles. Compared to detergent, the abundance of the high‐FRET peak has nearly doubled (29 %), indicating an increased population of occluded or outward‐open states. Indeed, when we now add N00 to block the opening of the periplasmic side, we find a significant reduction of the high‐FRET population (Figure 4 B), suggesting that a dominant portion of the high‐FRET peak represents outward‐open conformers.

Clearly, the cooperativity between the cytoplasmic and periplasmic side has been restored, thus making the outward‐open state accessible. Apparently, a lipid environment is required to sample outward‐open states. It is unclear, however, whether occluded states are also populated in POPE SapNPs. Yet, given the results of our experiments in detergent, we can identify them based on their rapid millisecond formation. Indeed, also in POPE SapNPs, we find open‐close kinetics with a decay time of 1 ms, irrespective of the presence of N00 (Figure 4 C), indicating a fast cytoplasmic opening and closing despite a locked periplasmic side. Occluded conformers are therefore found under all conditions whereas the outward‐open conformation is only populated in POPE containing SapNPs. This finding raises an important question: is a lipid bilayer *per se* sufficient to populate outward‐open ensembles or are special lipid head groups, e.g., ethanolamine in POPE required? To answer this question, we reconstituted DtpA in lipid environments of different composition (Figure 4 D–F): POPS (1‐palmitoyl‐2‐oleoyl‐sn‐glycero‐3‐phospho‐l‐serine), POPA (1‐palmitoyl‐2‐oleoyl‐sn‐glycero‐3‐phosphate), or BL (brain lipid) extract. Surprisingly, the FRET histograms differ strongly among the four membrane environments. In total, three populations can be distinguished. The high‐ and low‐FRET peaks are identical to those found in POPE. However, an additional peak with an extremely low FRET efficiency (*E*=0.25±0.02) is found in POPS and POPA (Figure 4 D,E) that corresponds to an ensemble with an extreme opening of the cytoplasmic side. In BL‐extracts on the contrary, neither high‐ nor low‐FRET peaks are observed and the transporter samples exclusively inward‐open conformations (Figure 4 F). Hence, DtpA exhibits a strong sensitivity to the lipid composition and net negatively charged lipids (POPS, POPA) even cause an extreme opening. Importantly, irrespective of the membrane composition, we find a strong cooperativity between the cytoplasmic and periplasmic side of the transporter: when N00 binds the periplasmic side, all populations collapse to predominantly form the inward‐open ensemble (Figure 4 B, D–F). The results indicate that it is the membrane environment *per se* that generates the cooperativity between the hemispheres of DtpA rather than the chemical nature of the lipid head group. Head group chemistry on the contrary, sensitively tunes the abundance of states. However, this is not the case for ligands. Similar to our findings in detergent, the addition of ligands to DtpA in POPE SapNPs did not affect the relative abundance of inward‐open and outward‐open states (SI Appendix, Figure S19). Although the detergent‐based inward‐open *x*‐ray structure with a pro‐drug bound is also similar to the apo‐structure,^48^ the lacking structural sensitivity to ligands is surprising. However, in cells, ligand concentrations differ significantly between periplasm and cytoplasm, thus generating an asymmetry that is not reproduced in our experiments. If ligand affinities are similar at each side of DtpA, the isotropic concentration of ligand on both sides in our experiments will cause a structural invariance of DtpA towards the ligand.

### Structural Modeling of DtpA Conformations

**Figure 5:**
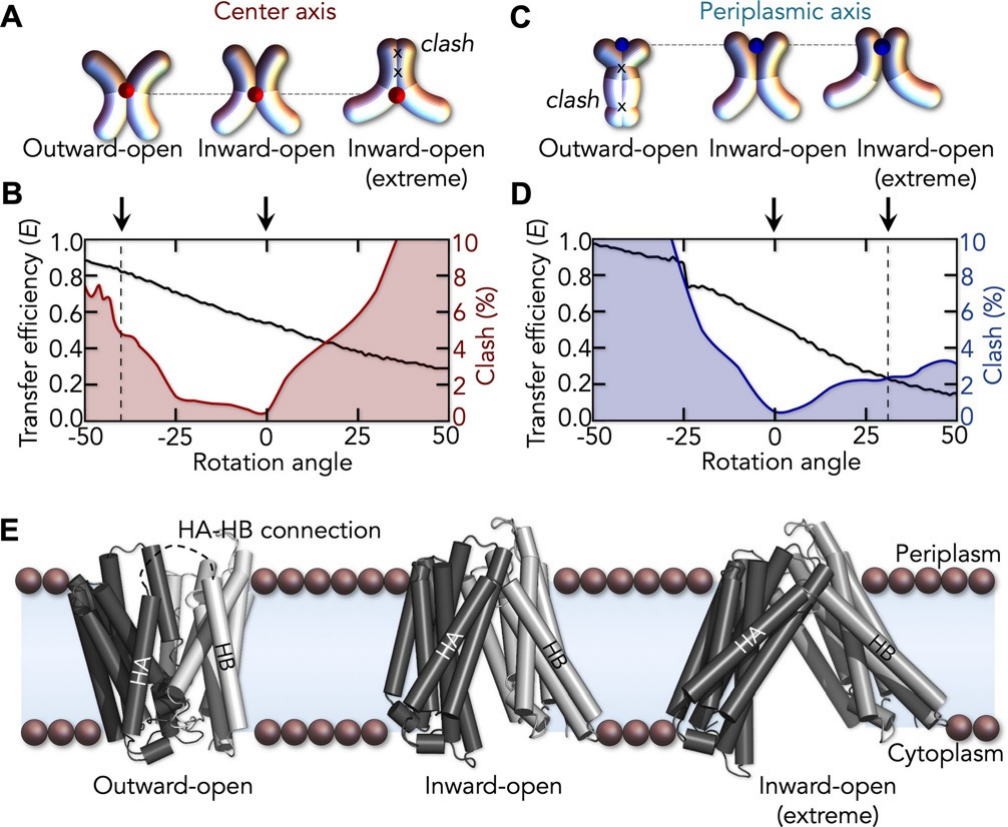
Structural models of the DtpA conformers. A) Schematics of DtpA rigid‐body rotations around the center axis. B) FRET efficiency (black) and clash score (red) for rotations around the center axis. C,D) Same as (A) and (B) for rotations around the periplasmic axis. E) Models of the outward‐open (left) and extreme inward‐open (right) states based on rigid‐body domain rotations of the X‐ray structure (middle).

The extreme inward‐open population observed in SapNPs containing POPS and POPA remains a conundrum. To obtain an estimate of the domain arrangement, we started from the existing *x*‐ray structure of DtpA and used rigid‐body rotations of the N‐ and C‐terminal domains (Figure 5 A–D). Current transport models^33^ suggest symmetric motions around the center axis that crosses the substrate‐binding site at the domain interface (Figure 5 A). We therefore rotated the domains around this axis and determined the FRET efficiency of the rotamers (see Methods in the Supporting Information). We find that closing the cytoplasmic side by rotating the domains by −40° reproduces the experimental FRET efficiency (0.82) of the outward‐open ensemble with only introducing 4.8 % atomic clashes^91^ (Figure 5 B).

However, it is impossible to match the experimental FRET efficiency of the extremely inward‐open ensemble without inacceptable clashes. We therefore chose an alternative rotation axis on the periplasmic side (Figure 5 C). Here, the extreme inward‐opening can indeed be reproduced with only 2.4 % atomic clashes (Figure 5 D). Notably, we did not optimize these structures, which shows that the domain architecture in principle allows an extreme cytoplasmic opening of DtpA (Figure 5 E).

Interestingly, like all bacterial POTs, DtpA contains two transmembrane helices (HA and HB) that are missing in eukaryotic homologs.^48^ The interaction between these helices and the core of DtpA hinders the formation of the outward‐open state via rigid body rotations and we therefore neglected the connecting loop between HA and HB in our rigid‐body motion approach (Figure 5 E). Neglecting these restraints is justified by the fact that the interface between both helices and DtpA is rich in hydrophobic residues. We conjecture that while these helices are strongly attached to DtpA in the more water‐rich detergent environment^25^ due to hydrophobic effects, this interaction is weakened in the strongly hydrophobic environment of a lipid bilayer. Weakening contacts between the helices and the DtpA core would release the restraints that hamper an opening of the periplasmic side such that this mechanism explains why the outward‐open state is only observed in membrane‐mimicking SapNPs (Figure 4).

## Conclusion

POTs have been extensively studied by *x*‐ray crystallography and biochemical transport assays over the past years.^27^, ^36^, ^39^, ^58^, ^61^, ^64^, ^92^, ^93^ Numerous structures of various bacterial homologues in the absence and presence of substrates, drugs, and prodrugs are available, highlighting crucial residues for substrate binding and proton coupling.^40^, ^41^, ^42^, ^43^, ^44^, ^45^, ^46^, ^47^, ^48^, ^49^, ^50^ Unfortunately, only inward‐open and partially inward‐open states of POTs have so far been described at atomic resolution in detergent and the influence of the membrane environment on structure and transport has been difficult to access.^40^, ^41^, ^43^, ^44^, ^46^, ^49^, ^54^ A number of reports showed that the activity of membrane proteins is preserved in detergent environments.^94^, ^95^, ^96^ Yet, counter examples are also frequent. For example, G‐protein coupled receptors (GPCRs) are known to be sensitive to the sterol content of lipid bilayers^97^ and their membrane extraction is often cell‐type dependent.^98^ Structural differences in a scramblase between detergent and membrane‐like environments have recently been found using cryo‐EM.^99^ Hence, the way lipid compositions affect structure and function of membrane proteins in vitro varies and it is often unclear whether a loss of function in detergent is caused by a loss in stability or by trapping the protein in one out of several functional conformers. While the latter is clearly preferred, it complicates the crystallization of all functionally relevant states. This is particularly problematic for POTs whose broad substrate profile makes them key for the pharmacokinetics of drugs. Using DtpA, we showed that the crystallization bias of POTs results from a strong sensitivity towards the lipid environment. Detergents do not allow a sampling of all functional states of DtpA. Although our smFRET experiments show that the inward‐open conformation in detergent is similar to that in POPE nanoparticles (Figure 2 A and B), the outward‐open state is only accessible in the more native environment of SapNPs. Structurally, a weakening of the hydrophobic contacts between the helices HA/HB and the core of DtpA in the low dielectric medium of a membrane explains this sensitivity (Figure 5 E). However, structural differences are not only observed between detergent and SapNPs but also between SapNPs with different lipid composition. In fact, since the aliphatic tails of POPA, POPS, and POPE are identical, the difference in the abundance of inward‐open, extreme inward‐open, and outward‐open conformers (Figure 4) is caused by the chemistry of lipid head groups. For example, the newly identified extreme inward‐open conformation is only observed in POPA and POPS, i.e., lipids with net negatively charged head groups. Given the complexity of natural membranes in which lipid compositions can vary on sub‐micrometer length scales, e.g., in micro‐domains,^100^ the heterogeneity found among four membrane‐like environments in our experiments may suggest a structurally heterogeneous DtpA ensemble in the cell membrane.

Care has to be taken when interpreting functional and pharmacological aspects based on structures obtained in detergents. While these structures suggest models with well‐defined states, our results sketch DtpA as an ensemble of multiple conformers with rapid dynamics. Recent studies showed that enzymes obey structural dynamics much faster than the timescales required for turnover.^101^, ^102^ Such dynamics are also prevalent in DtpA, irrespective of the lipid environment (Figure 3 C,D and Figure 4 C). Not only do inward‐open and occluded states interconvert rapidly with a relaxation time of 1 ms, but the inward‐open state itself is an ensemble of conformers within which the opening of the cytoplasmic side can vary (Figure 2 A). Although the role of these dynamics in the substrate transport cycle is currently elusive, fast domain closures in adenylate kinase have been proposed to optimize the orientation of substrate for catalysis^101^ and a similar mechanism could guide the POT‐mediated transport of peptides across biological membranes.

In summary, our results show that DtpA is a flexible and highly dynamic ensemble of structures that responds sensitively to its lipid environment and it will be important to understand whether this sensitivity is a general feature of MFS transporters.

## Conflict of interest

The authors declare no conflict of interest.

## Supporting information

As a service to our authors and readers, this journal provides supporting information supplied by the authors. Such materials are peer reviewed and may be re‐organized for online delivery, but are not copy‐edited or typeset. Technical support issues arising from supporting information (other than missing files) should be addressed to the authors.

Supplementary

## References

[1] R. R. Cowden , E. D. P. DeRobertis , E. M. F. DeRobertis , Cell and Molecular Biology, Lea & Febiger, Philadelphia, 1988.

[2] J. M. Scherrmann in Comprehensive Medicinal Chemistry II (Eds.: J. B. Taylor, D. J. Triggle), Elsevier, Oxford, 2006, pp. 51–85.

[3] I. Ubarretxena-Belandia , D. M. Engelman , Curr. Opin. Struct. Biol. 2001, 11, 370–376. 11406389 10.1016/s0959-440x(00)00217-7

[4] A. J. Rice , A. Park , H. W. Pinkett , Crit. Rev. Biochem. Mol. Biol. 2014, 49, 426–437. 25155087 10.3109/10409238.2014.953626 PMC4245157

[5] M. I. Borges-Walmsley , K. S. McKeegan , A. R. Walmsley , Biochem. J. 2003, 376, 313–338. 13678421 10.1042/BJ20020957 PMC1223791

[6] M. I. Borges-Walmsley , A. R. Walmsley , Trends Microbiol. 2001, 9, 71–79. 11173246 10.1016/s0966-842x(00)01920-x

[7] D. Hilger , M. Masureel , B. K. Kobilka , Nat. Struct. Mol. Biol. 2018, 25, 4–12. 29323277 10.1038/s41594-017-0011-7 PMC6535338

[8] E. Wallin , G. Von Heijne , Protein Sci. 1998, 7, 1029–1038. 9568909 10.1002/pro.5560070420 PMC2143985

[9] K. C. Chou , D. W. Elrod , Proteins Struct. Funct. Genet. 1999, 34, 137–153. 10336379

[10] J. Gong , Y. Chen , F. Pu , P. Sun , F. He , L. Zhang , Y. Li , Z. Ma , H. Wang , Curr. Drug Targets 2018, 20, 551–564. 10.2174/1389450120666181204164721 30516106

[11] J. P. Overington , B. Al-Lazikani , A. L. Hopkins , Nat. Rev. Drug Discovery 2006, 5, 993–996. 17139284 10.1038/nrd2199

[12] H. X. Zhou , T. A. Cross , Annu. Rev. Biophys. 2013, 42, 361–392. 23451886 10.1146/annurev-biophys-083012-130326 PMC3731949

[13] A. M. Seddon , P. Curnow , P. J. Booth , Biochim. Biophys. Acta Biomembr. 2004, 1666, 105–117. 10.1016/j.bbamem.2004.04.011 15519311

[14] W. Dowhan , M. Bogdanov , Biochem. Soc. Trans. 2011, 39, 767–774. 21599647 10.1042/BST0390767 PMC3348788

[15] C. Hunte , S. Richers , Curr. Opin. Struct. Biol. 2008, 18, 406–411. 18495472 10.1016/j.sbi.2008.03.008

[16] H. Palsdottir , C. Hunte , Biochim. Biophys. Acta Biomembr. 2004, 1666, 2–18. 10.1016/j.bbamem.2004.06.012 15519305

[17] C. Martens , M. Shekhar , A. J. Borysik , A. M. Lau , E. Reading , E. Tajkhorshid , P. J. Booth , A. Politis , Nat. Commun. 2018, 9, 4151. 30297844 10.1038/s41467-018-06704-1 PMC6175955

[18] J. U. Bowie , Curr. Opin. Struct. Biol. 2001, 11, 397–402. 11495729 10.1016/s0959-440x(00)00223-2

[19] K. Duquesne , V. Prima , J. N. Sturgis , Heterologous Expression of Membrane Proteins, Springer New York, New York, 2016.

[20] G. G. Privé , Methods 2007, 41, 388–397. 17367711 10.1016/j.ymeth.2007.01.007

[21] Z. Shao , J. Yin , K. Chapman , M. Grzemska , L. Clark , J. Wang , D. M. Rosenbaum , Nature 2016, 540, 602–606. 27851727 10.1038/nature20613 PMC5433929

[22] J. Payandeh , T. Scheuer , N. Zheng , W. A. Catterall , Nature 2011, 475, 353–359. 21743477 10.1038/nature10238 PMC3266868

[23] H. R. Schmidt , S. Zheng , E. Gurpinar , A. Koehl , A. Manglik , A. C. Kruse , Nature 2016, 532, 527–530. 27042935 10.1038/nature17391 PMC5550834

[24] Y. Sonoda , S. Newstead , N. J. Hu , Y. Alguel , E. Nji , K. Beis , S. Yashiro , C. Lee , J. Leung , A. D. Cameron , B. Byrne , S. Iwata , D. Drew , Structure 2011, 19, 17–25. 21220112 10.1016/j.str.2010.12.001 PMC3111809

[25] C. Chipot , F. Dehez , J. R. Schnell , N. Zitzmann , E. Pebay-Peyroula , L. J. Catoire , B. Miroux , E. R. S. Kunji , G. Veglia , T. A. Cross , P. Schanda , Chem. Rev. 2018, 118, 3559–3607. 29488756 10.1021/acs.chemrev.7b00570 PMC5896743

[26] A. Stetsenko , A. Guskov , Crystals 2017, 7, 197.

[27] G. P. Rédei , Encyclopedia of Genetics, Genomics, Proteomics, and Informatics, Vol. 62, Springer, Heidelberg, 2008, pp. 1142-1142.

[28] V. S. Reddy , M. A. Shlykov , R. Castillo , E. I. Sun , M. H. Saier , FEBS J. 2012, 279, 2022–2035. 22458847 10.1111/j.1742-4658.2012.08588.x PMC3425384

[29] N. Yan , Annu. Rev. Biophys. 2015, 44, 257–283. 26098515 10.1146/annurev-biophys-060414-033901

[30] O. Jardetzky , Nature 1966, 211, 969–970. 5968307 10.1038/211969a0

[31] J. Abramson , I. Smirnova , V. Kasho , G. Verner , H. R. Kaback , S. Iwata , Science 2003, 301, 610–615. 12893935 10.1126/science.1088196

[32] L. R. Forrest , R. Krämer , C. Ziegler , Biochim. Biophys. Acta Bioenerg. 2011, 1807, 167–188. 10.1016/j.bbabio.2010.10.014 21029721

[33] E. M. Quistgaard , C. Löw , F. Guettou , P. Nordlund , Nat. Rev. Mol. Cell Biol. 2016, 17, 123–132. 26758938 10.1038/nrm.2015.25

[34] M. D. Marger , M. H. Saier , Trends Biochem. Sci. 1993, 18, 13–20. 8438231 10.1016/0968-0004(93)90081-w

[35] I. Rubio-Aliaga , H. Daniel , Xenobiotica 2008, 38, 1022–1042. 18668438 10.1080/00498250701875254

[36] M. Brandsch , Expert Opin. Drug Metab. Toxicol. 2009, 5, 887–905. 19519280 10.1517/17425250903042292

[37] K. M. Giacomini , S. M. Huang , D. J. Tweedie , L. Z. Benet , K. L. R. Brouwer , X. Chu , A. Dahlin , R. Evers , V. Fischer , K. M. Hillgren , K. A. Hoffmaster , T. Ishikawa , D. Keppler , R. B. Kim , C. A. Lee , M. Niemi , J. W. Polli , Y. Sugiyama , P. W. Swaan , J. A. Ware , S. H. Wright , S. Wah Yee , M. J. Zamek-Gliszczynski , L. Zhang , Nat. Rev. Drug Discovery 2010, 9, 215–236. 20190787 10.1038/nrd3028 PMC3326076

[38] A. H. Dantzig , L. Bergin , Biochim. Biophys. Acta Biomembr. 1990, 1027, 211–217. 10.1016/0005-2736(90)90309-c 2397233

[39] A. Biegel , S. Gebauer , B. Hartrodt , M. Brandsch , K. Neubert , I. Thondorf , J. Med. Chem. 2005, 48, 4410–4419. 15974593 10.1021/jm048982w

[40] R. Boggavarapu , J. M. Jeckelmann , D. Harder , Z. Ucurum , D. Fotiadis , BMC Biol. 2015, 13, 58. 26246134 10.1186/s12915-015-0167-8 PMC4527243

[41] S. Doki , H. E. Kato , N. Solcan , M. Iwaki , M. Koyama , M. Hattori , N. Iwase , T. Tsukazaki , Y. Sugita , H. Kandori , S. Newstead , R. Ishitani , O. Nureki , Proc. Natl. Acad. Sci. USA 2013, 110, 11343–11348. 23798427 10.1073/pnas.1301079110 PMC3710879

[42] G. S. Minhas , S. Newstead , Proc. Natl. Acad. Sci. USA 2019, 116, 804–809. 30602453 10.1073/pnas.1813715116 PMC6338836

[43] S. Newstead , D. Drew , A. D. Cameron , V. L. G. Postis , X. Xia , P. W. Fowler , J. C. Ingram , E. P. Carpenter , M. S. P. Sansom , M. J. McPherson , S. A. Baldwin , S. Iwata , EMBO J. 2011, 30, 417–426. 21131908 10.1038/emboj.2010.309 PMC3025455

[44] J. L. Parker , C. Li , A. Brinth , Z. Wang , L. Vogeley , N. Solcan , G. Ledderboge-Vucinic , J. M. J. Swanson , M. Caffrey , G. A. Voth , S. Newstead , Proc. Natl. Acad. Sci. USA 2017, 114, 13182–13187. 29180426 10.1073/pnas.1710727114 PMC5740623

[45] J. L. Parker , S. Newstead , Nature 2014, 507, 68–72. 24572366 10.1038/nature13116 PMC3982047

[46] E. M. Quistgaard , M. M. Molledo , C. Löw , PLoS One 2017, 12, e0173126. 28264013 10.1371/journal.pone.0173126 PMC5338821

[47] N. Solcan , J. Kwok , P. W. Fowler , A. D. Cameron , D. Drew , S. Iwata , S. Newstead , EMBO J. 2012, 31, 3411–3421. 22659829 10.1038/emboj.2012.157 PMC3419923

[48] Y. Ural-Blimke , A. Flayhan , J. Strauss , V. Rantos , K. Bartels , R. Nielsen , E. Pardon , J. Steyaert , J. Kosinski , E. M. Quistgaard , C. Löw , J. Am. Chem. Soc. 2019, 141, 2404–2412. 30644743 10.1021/jacs.8b11343

[49] Y. Zhao , G. Mao , M. Liu , L. Zhang , X. Wang , X. C. Zhang , Structure 2014, 22, 1152–1160. 25066136 10.1016/j.str.2014.06.008

[50] P. W. Fowler , M. Orwick-Rydmark , S. Radestock , N. Solcan , P. M. Dijkman , J. A. Lyons , J. Kwok , M. Caffrey , A. Watts , L. R. Forrest , S. Newstead , Structure 2015, 23, 290–301. 25651061 10.1016/j.str.2014.12.012 PMC4321885

[51] F. Guettou , E. M. Quistgaard , M. Raba , P. Moberg , C. Löw , P. Nordlund , Nat. Struct. Mol. Biol. 2014, 21, 728–731. 25064511 10.1038/nsmb.2860

[52] F. Guettou , E. M. Quistgaard , L. Trésaugues , P. Moberg , C. Jegerschöld , L. Zhu , A. J. O. Jong , P. Nordlund , C. Löw , EMBO Rep. 2013, 14, 804–810. 23867627 10.1038/embor.2013.107 PMC3790050

[53] C. Y. Huang , V. Olieric , P. Ma , N. Howe , L. Vogeley , X. Liu , R. Warshamanage , T. Weinert , E. Panepucci , B. Kobilka , K. Diederichs , M. Wang , M. Caffrey , Acta Crystallogr. Sect. D 2016, 72, 93–112. 10.1107/S2059798315021683 PMC4756617 26894538

[54] J. A. Lyons , J. L. Parker , N. Solcan , A. Brinth , D. Li , S. T. Shah , M. Caffrey , S. Newstead , EMBO Rep. 2014, 15, 886–893. 24916388 10.15252/embr.201338403 PMC4149780

[55] M. Martinez Molledo , E. M. Quistgaard , A. Flayhan , J. Pieprzyk , C. Löw , Structure 2018, 26, 467–476.e4. 29429879 10.1016/j.str.2018.01.005 PMC5845931

[56] M. Martinez Molledo , E. M. Quistgaard , C. Löw , FEBS Lett. 2018, 592, 3239–3247. 30194725 10.1002/1873-3468.13246 PMC6221056

[57] G. S. Minhas , D. Bawdon , R. Herman , M. Rudden , A. P. Stone , A. G. James , G. H. Thomas , S. Newstead , eLife 2018, 7, e34995. 29966586 10.7554/eLife.34995 PMC6059767

[58] D. Harder , J. Stolz , F. Casagrande , P. Obrdlik , D. Weitz , D. Fotiadis , H. Daniel , FEBS J. 2008, 275, 3290–3298. 18485005 10.1111/j.1742-4658.2008.06477.x

[59] C. A. Bippes , L. Ge , M. Meury , D. Harder , Z. Ucurum , H. Daniel , D. Fotiadis , D. J. Müller , Proc. Natl. Acad. Sci. USA 2013, 110, 3978–3986. 10.1073/pnas.1312959110 PMC3801073 24082128

[60] H. Daniel , B. Spanier , G. Kottra , D. Weitz , Physiology 2006, 21, 93–102. 16565475 10.1152/physiol.00054.2005

[61] M. Brandsch , Curr. Opin. Pharmacol. 2013, 13, 881–887. 24007794 10.1016/j.coph.2013.08.004

[62] M. Brandsch , I. Knütter , F. H. Leibach , Eur. J. Pharm. Sci. 2004, 21, 53–60. 14706811 10.1016/s0928-0987(03)00142-8

[63] S. M. Ocheltree , H. Shen , Y. Hu , R. F. Keep , D. E. Smith , J. Pharmacol. Exp. Ther. 2005, 315, 240–247. 15987832 10.1124/jpet.105.089359

[64] D. Weitz , D. Harder , F. Casagrande , D. Fotiadis , P. Obrdlik , B. Kelety , H. Daniel , J. Biol. Chem. 2007, 282, 2832–2839. 17158458 10.1074/jbc.M604866200

[65] B. K. Prabhala , N. G. Aduri , M. Iqbal , M. Rahman , M. Gajhede , P. R. Hansen , O. Mirza , Res. Microbiol. 2017, 168, 443–449. 28214542 10.1016/j.resmic.2017.01.005

[66] J. H. Beale , J. L. Parker , F. Samsudin , A. L. Barrett , A. Senan , L. E. Bird , D. Scott , R. J. Owens , M. S. P. Sansom , S. J. Tucker , D. Meredith , P. W. Fowler , S. Newstead , Structure 2015, 23, 1889–1899. 26320580 10.1016/j.str.2015.07.016 PMC4597091

[67] Y. Ashok , R. Nanekar , V. P. Jaakola , Protein Eng. Des. Sel. 2015, 28, 539–542. 26384510 10.1093/protein/gzv049

[68] D. M. Rosenbaum , C. Zhang , J. A. Lyons , R. Holl , D. Aragao , D. H. Arlow , S. G. F. Rasmussen , H. J. Choi , B. T. Devree , R. K. Sunahara , P. S. Chae , S. H. Gellman , R. O. Dror , D. E. Shaw , W. I. Weis , M. Caffrey , P. Gmeiner , B. K. Kobilka , Nature 2011, 469, 236–242. 21228876 10.1038/nature09665 PMC3074335

[69] J. F. White , N. Noinaj , Y. Shibata , J. Love , B. Kloss , F. Xu , J. Gvozdenovic-Jeremic , P. Shah , J. Shiloach , C. G. Tate , R. Grisshammer , Nature 2012, 490, 508–513. 23051748 10.1038/nature11558 PMC3482300

[70] K. H. Cho , H. E. Bae , M. Das , S. H. Gellman , P. S. Chae , Chem. Asian J. 2014, 9, 632–638. 24288216 10.1002/asia.201301303

[71] S. Granier , A. Manglik , A. C. Kruse , T. S. Kobilka , F. S. Thian , W. I. Weis , B. K. Kobilka , Nature 2012, 485, 400–404. 22596164 10.1038/nature11111 PMC3523198

[72] K. Haga , A. C. Kruse , H. Asada , T. Yurugi-Kobayashi , M. Shiroishi , C. Zhang , W. I. Weis , T. Okada , B. K. Kobilka , T. Haga , T. Kobayashi , Nature 2012, 482, 547–551. 22278061 10.1038/nature10753 PMC3345277

[73] A. C. Kruse , J. Hu , A. C. Pan , D. H. Arlow , D. M. Rosenbaum , E. Rosemond , H. F. Green , T. Liu , P. S. Chae , R. O. Dror , D. E. Shaw , W. I. Weis , J. Wess , B. K. Kobilka , Nature 2012, 482, 552–556. 22358844 10.1038/nature10867 PMC3529910

[74] A. Manglik , A. C. Kruse , T. S. Kobilka , F. S. Thian , J. M. Mathiesen , R. K. Sunahara , L. Pardo , W. I. Weis , B. K. Kobilka , S. Granier , Nature 2012, 485, 321–326. 22437502 10.1038/nature10954 PMC3523197

[75] S. G. F. Rasmussen , B. T. Devree , Y. Zou , A. C. Kruse , K. Y. Chung , T. S. Kobilka , F. S. Thian , P. S. Chae , E. Pardon , D. Calinski , J. M. Mathiesen , S. T. A. Shah , J. A. Lyons , M. Caffrey , S. H. Gellman , J. Steyaert , G. Skiniotis , W. I. Weis , R. K. Sunahara , B. K. Kobilka , Nature 2011, 477, 549–557. 21772288 10.1038/nature10361 PMC3184188

[76] A. M. Ring , A. Manglik , A. C. Kruse , M. D. Enos , W. I. Weis , K. C. Garcia , B. K. Kobilka , Nature 2013, 502, 575–579. 24056936 10.1038/nature12572 PMC3822040

[77] S. E. Rollauer , M. J. Tarry , J. E. Graham , M. Jääskeläinen , F. Jäger , S. Johnson , M. Krehenbrink , S. M. Liu , M. J. Lukey , J. Marcoux , M. A. McDowell , F. Rodriguez , P. Roversi , P. J. Stansfeld , C. V. Robinson , M. S. P. Sansom , T. Palmer , M. Högbom , B. C. Berks , S. M. Lea , Nature 2012, 492, 210–214. 23201679 10.1038/nature11683 PMC3573685

[78] I. V. Gopich , A. Szabo , Proc. Natl. Acad. Sci. USA 2012, 109, 7747–7752. 22550169 10.1073/pnas.1205120109 PMC3356627

[79] S. Kalinin , A. Valeri , M. Antonik , S. Felekyan , C. A. M. Seidel , J. Phys. Chem. B 2010, 114, 7983–7995. 20486698 10.1021/jp102156t

[80] A. Soranno , B. Buchli , D. Nettels , R. R. Cheng , S. Müller-Späth , S. H. Pfeil , A. Hoffmann , E. A. Lipman , D. E. Makarov , B. Schuler , Proc. Natl. Acad. Sci. USA 2012, 109, 17800–17806. 22492978 10.1073/pnas.1117368109 PMC3497802

[81] E. Haas , M. Wilchek , E. Katchalski-Katzir , I. Z. Steinberg , Proc. Natl. Acad. Sci. USA 1975, 72, 1807–1811. 1057171 10.1073/pnas.72.5.1807 PMC432635

[82] R. Wuttke , H. Hofmann , D. Nettels , M. B. Borgia , J. Mittal , R. B. Best , B. Schuler , Proc. Natl. Acad. Sci. USA 2014, 111, 5213–5218. 24706910 10.1073/pnas.1313006111 PMC3986154

[83] D. Nettels , S. Müller-Späth , F. Küster , H. Hofmann , D. Haenni , S. Rüegger , L. Reymond , A. Hoffmann , J. Kubelka , B. Heinz , K. Gast , R. B. Best , B. Schuler , Proc. Natl. Acad. Sci. USA 2009, 106, 20740–20745. 19933333 10.1073/pnas.0900622106 PMC2791578

[84] A. Hoffmann , D. Nettels , J. Clark , A. Borgia , S. E. Radford , J. Clarke , B. Schuler , Phys. Chem. Chem. Phys. 2011, 13, 1857–1871. 21218223 10.1039/c0cp01911a PMC3378030

[85] I. Grossman , H. Y. Aviram , G. Armony , A. Horovitz , H. Hofmann , G. Haran , D. Fass , Nat. Commun. 2015, 6, 8624. 26468675 10.1038/ncomms9624 PMC4634331

[86] X. Jiang , N. Ermolova , J. Lim , S. W. Choi , H. R. Kaback , Proc. Natl. Acad. Sci. USA 2020, 117, 977–981. 31889006 10.1073/pnas.1916563117 PMC6969543

[87] A. Flayhan , H. D. T. Mertens , Y. Ural-Blimke , M. Martinez Molledo , D. I. Svergun , C. Löw , Structure 2018, 26, 345–355.e5. 29413323 10.1016/j.str.2018.01.007 PMC5807053

[88] J. Frauenfeld , R. Löving , J. P. Armache , A. F. P. Sonnen , F. Guettou , P. Moberg , L. Zhu , C. Jegerschöld , A. Flayhan , J. A. G. Briggs , H. Garoff , C. Löw , Y. Cheng , P. Nordlund , Nat. Methods 2016, 13, 345–351. 26950744 10.1038/nmeth.3801 PMC4894539

[89] D. Oursel , C. Loutelier-Bourhis , N. Orange , S. Chevalier , V. Norris , C. M. Lange , Rapid Commun. Mass Spectrom. 2007, 21, 1721–1728. 17477452 10.1002/rcm.3013

[90] G. Van Meer , D. R. Voelker , G. W. Feigenson , Nat. Rev. Mol. Cell Biol. 2008, 9, 112–124. 18216768 10.1038/nrm2330 PMC2642958

[91] S. Gore , E. Sanz García , P. M. S. Hendrickx , A. Gutmanas , J. D. Westbrook , H. Yang , Z. Feng , K. Baskaran , J. M. Berrisford , B. P. Hudson , Y. Ikegawa , N. Kobayashi , C. L. Lawson , S. Mading , L. Mak , A. Mukhopadhyay , T. J. Oldfield , A. Patwardhan , E. Peisach , G. Sahni , M. R. Sekharan , S. Sen , C. Shao , O. S. Smart , E. L. Ulrich , R. Yamashita , M. Quesada , J. Y. Young , H. Nakamura , J. L. Markley , H. M. Berman , S. K. Burley , S. Velankar , G. J. Kleywegt , Structure 2017, 25, 1916–1927. 29174494 10.1016/j.str.2017.10.009 PMC5718880

[92] H. A. Ernst , A. Pham , H. Hald , J. S. Kastrup , M. Rahman , O. Mirza , Biochem. Biophys. Res. Commun. 2009, 389, 112–116. 19703419 10.1016/j.bbrc.2009.08.098

[93] J. L. Parker , J. A. Mindell , S. Newstead , eLife 2014, 3, e04273. 10.7554/eLife.04273 PMC4271188 25457052

[94] M. J. Berardi , W. M. Shih , S. C. Harrison , J. J. Chou , Nature 2011, 476, 109–114. 21785437 10.1038/nature10257 PMC3150631

[95] L. Zhao , S. Wang , Q. Zhu , B. Wu , Z. Liu , B. OuYang , J. J. Chou , Structure 2017, 25, 1371–1379.e3. 28781081 10.1016/j.str.2017.07.005

[96] R. Matar-Merheb , M. Rhimi , A. Leydier , F. Huché , C. Galián , E. Desuzinges-Mandon , D. Ficheux , D. Flot , N. Aghajari , R. Kahn , A. Di Pietro , J. M. Jault , A. W. Coleman , P. Falson , PLoS One 2011, 6, e18036. 21483854 10.1371/journal.pone.0018036 PMC3069034

[97] M. Casiraghi , M. Damian , E. Lescop , E. Point , K. Moncoq , N. Morellet , D. Levy , J. Marie , E. Guittet , J. L. Banères , L. J. Catoire , J. Am. Chem. Soc. 2016, 138, 11170–11175. 27489943 10.1021/jacs.6b04432

[98] J. Thomas , C. G. Tate , J. Mol. Biol. 2014, 426, 4139–4154. 25454020 10.1016/j.jmb.2014.10.012 PMC4271737

[99] V. Kalienkova , V. C. Mosina , L. Bryner , G. T. Oostergetel , R. Dutzler , C. Paulino , eLife 2019, 8, e44364. 30785398 10.7554/eLife.44364 PMC6414200

[100] B. A. Truong-Quang , P. F. Lenne , Front. Plant Sci. 2014, 5, 18. 24600455 10.3389/fpls.2014.00018 PMC3927121

[101] H. Y. Aviram , M. Pirchi , H. Mazal , Y. Barak , I. Riven , G. Haran , Proc. Natl. Acad. Sci. USA 2018, 115, 3243–3248. 29531052 10.1073/pnas.1720448115 PMC5879700

[102] I. Grossman-Haham , G. Rosenblum , T. Namani , H. Hofmann , Proc. Natl. Acad. Sci. USA 2018, 115, 513–518. 29298911 10.1073/pnas.1714401115 PMC5776979

