# Supplementary material for: Membrane Chemistry Tunes the Structure of a Peptide Transporter
Source: Angew Chem Int Ed Engl. 2020 Sep 11;59(43):19121–8. doi: 10.1002/anie.202008226 (PMC7590137; doi:10.1002/anie.202008226)
Supplement: Supplementary file 1 — Supplementary [file ANIE-59-19121-s001.pdf]

Angewandte Chemie
Eine Zeitschrift der Gesellschaft Deutscher Chemiker

Supporting Information

Membrane Chemistry Tunes the Structure of a Peptide Transporter**

Tanya Lasitza-Male[+], Kim Bartels[+], Jakub Jungwirth, Felix Wiggers, Gabriel Rosenblum, Hagen Hofmann, and Christian Löw**

anie_202008226_sm_miscellaneous_information.pdf

Table of Contents

Experimental Procedures 2
Results 7
References 16
Authors Contributions 17

Experimental Procedures

Chemicals. Unless specified otherwise, chemicals were purchased from Sigma-Aldrich, phospholipids and membrane extracts from Avanti Polar Lipids, Inc., n-dodecyl-ß-D-maltoside (DDM) and lauryl-maltose-neopentyl-glycol (LMNG) from Anatrace, antibiotics, iso-propyl-ß-d-thiogalactopyranoside (IPTG) and 1,4-dithiothreitol (DTT) from Roth, DNase I from Appli-Chem, Lysozyme and protease inhibitor cocktail from Roche, di/tripeptides were purchased from Sigma-Aldrich, Fluka, and Bachem, Alexa Fluor 488 C5 maleimide and Alexa Fluor 594 C5 maleimide from ThermoFisher and Terrific broth (TB) from Melford.

Protein constructs. DtpA variants (Uniprot ID: P77304) were cloned into the pNIC-CTHF vector (Addgene plasmid-ID Plasmid #26105) using LIC cloning [1]. Point-mutations for FRET labeling variants were generated by site-directed mutagenesis. Briefly, wildtype DtpA in pNIC-CTHF vector was used as template for PCR with mutagen primers containing the desired mutation (SI Appendix, Table S1). Mutations were confirmed by DNA sequencing. Saposin A was cloned into the pNIC-Bsa4 expression vector (Addgene plasmid-ID Plasmid #26103) using LIC cloning. The nanobody (N00) was cloned into a pMESy2 vector.

DtpA variants, expression, purification and labeling. DtpA variants were expressed and purified as previously described [2]. The constructs were transformed into *E. coli* strain C41 (DE3) and grown in TB medium supplemented with 30 µg/ml kanamycin at 37°C. The cells were induced by 0.2 mM IPTG at OD_{600nm} of 0.6, incubated further for 16 hours at 18°C, and harvested by centrifugation (9,379 g, 15 min, 4°C). Then the cells were re-suspended in lysis buffer (20 mM sodium phosphate pH 7.5, 300 mM NaCl, 5% (v/v) glycerol, 15 mM imidazole, 0.5 mM TCEP, 5 units/ml of DNaseI, 1 mg/ml lysozyme, and protease inhibitor). Typically, 5 ml of lysis buffer were used for 1 g of cell pellet. Cells were lysed by three cycles using an EmulsiFlex-C3 (Avestin) at 10,000 psi. After a low-speed centrifugation step (10,000 g, 15 min, 4°C) that separated the undisrupted cells and debris, an ultracentrifugation step of the supernatant was performed to pellet the membrane fraction (142,400 g, 50 min, 4°C). The crude membranes were resuspended in lysis buffer, supplemented with 1% LMNG, 0.5 mM TCEP and protease inhibitor, and stirred for 60 min at 4°C. After an additional ultracentrifugation step (104,600 g, 50 min, 4°C), DtpA was purified by Immobilized Metal Affinity Chromatography (IMAC) using Ni-NTA agarose (ThermoFisher). His-tagged proteins were bound to the resin for 60 min at 4°C on a rotating wheel, extensively washed with increasing imidazole concentration (20 mM sodium phosphate pH 7.5, 300 mM NaCl, 5% (v/v) glycerol, 15-25 mM imidazole, 0.01% (w/v) LMNG, 0.5 mM TCEP) and eluted in elution buffer (20 mM sodium phosphate pH 7.5, 150 mM NaCl, 5% (v/v) glycerol, 250 mM imidazole, 0.01% (w/v) LMNG, 0.5 mM TCEP). The eluate was concentrated to 1 ml using a 50 MWCO concentrator (Corning Spin-X UF concentrators) and incubated with 10 mM DTT for 30 min at 4°C. Size exclusion chromatography (SEC) was performed on an ÄKTA Pure system (GE Healthcare Life Sciences) using a Superdex 200 Increase 10/300 GL column (GE Healthcare Life Sciences) equilibrated with SEC Buffer (20 mM sodium phosphate pH 7.5, 150 mM NaCl, 5% (v/v) glycerol, 0.01% (w/v) LMNG). Fractions containing the protein were pooled and concentrated to 500 µl. For labeling, the variants were incubated for 2 hours at RT under gentle agitation with a 1:1 mix of Alexa Fluor 488 C5 maleimide and Alexa Fluor 594 C5 maleimide dyes (ThermoFisher) in a 1:2.4 molar ratio of protein to dyes. To stop the labeling reaction, the samples were incubated with 1 mM L-Glutathione for 30 min at RT. Free dyes were removed using a PD-10 desalting column (GE Healthcare Life Sciences). An additional IMAC was performed, the eluate from the desalting column was incubated with Ni-NTA agarose for 60 min at 4°C on a rotating wheel, extensively washed with increasing imidazole concentration, and eluted in elution buffer. TEV protease was added to the eluate (0.3 mg TEV for material derived from a 0.5 l culture). The sample was dialyzed overnight at 4°C against SEC buffer. Negative IMAC was performed to recover the cleaved labeled variants. Protein containing fractions were loaded on SEC Superdex 200 Increase 10/300 GL column (GE Healthcare Life Sciences) equilibrated with SEC buffer. Fractions containing the protein were pooled and concentrated.

Nanobody (N00) expression and purification. The nanobody selection was previously described [2]. The nanobody expression plasmid was transformed into the *E. coli* strain WK6. The cells were grown at 37°C in TB medium supplemented with 100 µg/ml carbenicillin. At an OD_{600nm} of 0.7 the cells were induced with 1 mM IPTG and incubated for 16 h at 27°C. Cells were harvested (9,379 g, 15 min, 4°C). Afterwards, the cells were resuspended in 5 ml TES buffer (200 mM Tris pH 8.0, 0.5 mM EDTA, 500 mM sucrose) per 1 g of pellet. Fourfold diluted TES buffer was added to perform an osmotic shock. Cell debris were removed by centrifugation (10,000 g, 15 min, 4°C) and the supernatant was recovered and applied on a Capture Select column (ThermoFisher), equilibrated previously with wash buffer (20 mM sodium phosphate pH 7.5, 150 mM NaCl), and the protein was eluted in the elution buffer (20 mM sodium phosphate pH 7.5, 150 mM NaCl, 2 M $MgCl_2$). The eluted fractions were loaded on SEC HiLoad 16/600 Superdex 75 pg column (GE Healthcare Life Sciences), equilibrated with Nanobody wash buffer. Fractions containing protein were pooled and concentrated using a 5 MWCO concentrator (Corning Spin-X UF concentrators).

Saposin A (SapA) expression and purification. The SapA expression plasmid was transformed into the *E. coli* strain Rosetta gami-2(DE3). Transformed bacteria were grown at 37°C in TB medium supplemented with 30 µg/ml kanamycin, 34 µg/ml chloramphenicol and 10 µg/ml tetracycline. At an OD_{600nm} of 0.7, the cells were induced with 1 mM IPTG and incubated for 4 h at 37°C. Cells were harvested by centrifugation (9,379 g, 15 min, 4°C). Typically, 1 g of pellet was suspended in 5 ml lysis buffer (20 mM sodium phosphate pH 7.5, 300 mM NaCl, 5% (v/v) glycerol, 15 mM imidazole, supplemented with 5 units/ml DNaseI, 1 mg/ml lysozyme and protease inhibitor). Cells were lysed by three cycles using an EmulsiFlex-C3 (Avestin) at 10.000 psi. The lysate was incubated at 80°C, 10 min. Non-lysed cells, debris, and aggregated proteins were removed by centrifugation (10,000 g, 15 min, 4°C). The supernatant was purified by IMAC using Ni-NTA agarose. His-tagged samples were bound to the resin for 60 min at 4°C on a rotating wheel, extensively washed (20 mM sodium phosphate pH 7.5, 300 mM NaCl, 5% (v/v) glycerol, 15-30 mM imidazole), and eluted in an elution buffer (20 mM sodium phosphate pH 7.5, 150 mM NaCl, 5% (v/v) glycerol, 400 mM imidazole). TEV protease was added to the eluate and 2 mg were used to cleave protein resulting from 3 l culture. The sample was dialyzed overnight at 4°C against Saposin SEC Buffer (20 mM sodium phosphate pH 7.5, 150 mM NaCl). Negative IMAC was performed to recover cleaved protein. The last purification step was SEC on a HiLoad 16/600 Superdex 75 pg column equilibrated with Saposin SEC Buffer. Fraction containing protein were pooled, and concentrated using a 5 MWCO concentrator (Corning Spin-X UF concentrators).

Cysteine accessibility assay. We tested the accessibility of cysteine residues of DtpA variants before and after labeling. Briefly, 0.15 mg/m of each variant were incubated with methoxypolyethylene glycol maleimide 5000 (PEG-maleimide) (Sigma-Aldrich) at 2 mM final PEG-maleimide concentration for 30 min at RT. SDS-PAGE was performed using a 4-12% Bis-Tris gel (Expedeon) and stained using InstantBlue Coomassie Protein Stain (Expedeon) (SI Appendix, Fig. S9).

***In vivo* uptake assay.** The uptake assay of the fluorescent N-7-amino-4-methylcoumarin-3-acetic acid coupled to β-Ala-Lys (AK-AMCA) dipeptide was performed as described previously with minor changes [3]. For *in vivo* uptake, the DtpA expression plasmid was transformed into *E. coli* strain C41 (DE3). Transformed bacteria were grown at 37°C in TB medium supplemented with 30 µg/ml kanamycin. At an OD_{600nm} of 0.6, cells were induced with 0.2 mM IPTG and incubated for further 3 h at 37°C. Cells were harvested by centrifugation at 3,214 g for 15 min at 4°C and suspended in assay buffer (20 mM sodium phosphate pH 7.5, 150 mM NaCl, 5 mM glucose) to OD_{600nm} of 10. In a final volume of 100 µl, 50 µl assay buffer, and 40 µl cells at OD_{600nm} of 10 were incubated with 10 µl of 1 mM AK-AMCA for 20 min at 37°C. For the negative control, double distilled water instead of AK-AMCA was added. The reaction was stopped by the addition of 200 µl ice-cold assay buffer. Cells were washed twice with 200 µl ice-cold assay buffer and finally suspended in 200 µl ice-cold assay buffer. Remaining fluorescence was measured using a Tecan Spark multimode microplate reader (Tecan Life Sciences) with an excitation of 350 nm and emission of 450 nm. To correct for the number of cells contributing to the fluorescence signal, the OD_{600nm} for each sample was measured. Western blot was performed to account for differences in expression level of the variants and the wildtype. In short, SDS-PAGE using a 4-12% Bis-Tris gel (Expedeon) was performed and transferred on a PVDF membrane (Biorad). 2% BSA in TBST (Sigma) was used for blocking, TBST was used as washing buffer. The membrane was incubated with HisProbe-HRP conjugates antibody (ThermoFisher) for 1 h at RT. The blot was developed using Super Signal West Pico Substrate (ThermoFisher) and Super Signal West Femto Substrate (ThermoFisher) in a 1:10 ratio.

Reconstitution of DtpA variants in Saposin A nanoparticles (SapNPs). Labeled DtpA variants were reconstituted into SapNPs using a 1:20:35 molar ratio of DtpA variant:SapA:lipids [4,5]. Lipid stocks were prepared as previously described [5]. We used: 1-palmitoyl-2-oleoyl-sn-glycero-3-phospho-L-serine (POPS), 1-palmitoyl-2-oleoyl-sn-glycero-3-phosphoethanol-amine (POPE), 1-palmitoyl-2-oleoyl-sn-glycero-3-phosphate (POPA) and Brain Lipids Extract (Avanti Polar Lipids). Lipids were incubated at 37°C for 10 min, DtpA variant was added and incubated at RT for 15 min. After addition of SapA, the sample was incubated at RT for 20 min. Biobeads (Biorad) were added and the sample was incubated overnight at 4°C on a rotating wheel. The reconstituted DtpA was recovered by SEC Superdex 200 Increase 10/300 GL column (GE Healthcare), equilibrated with SEC buffer (20 mM sodium phosphate pH 7.5, 150 mM NaCl, 5% (v/v) glycerol). We confirmed the assembly by SDS-PAGE (SI Appendix, Fig. S15).

Thermal shift assay. The stability of DtpA was monitored by nanoDSF. 0.5 mg/ml of DtpA and its variants were incubated with the transporter ligands [2]: L-alanine-L-leucine (AL), L-alanine-L-phenylalanine (AF), L-alanine-L-phenylalanine (AF) and L-alanine-L-phenylalanine-L-alanine (AFA) at 2.5 mM final ligand concentration for 10 min at RT. In addition, DtpA at 0.5 mg/ml was incubated with N00 at a molar ratio of 1:1.2 for 10 min at RT. Standard grade nanoDSF capillaries (Nanotemper) were loaded into a Prometheus NT.48 device (Nanotemper) controlled by PR. ThermControl (version 2.1.2). Excitation power was adjusted to 20% and samples were heated from 20°C to 90°C with a slope of 1°C/min. All samples were examined in triplicates and error bars represent standard deviations.

Biolayer interferometry. Dissociation constants (K_D) of N00 binding to DtpA were measured using an Octet RED96 System (fortéBIO). To this end, N00 was biotinylated using an EZ-Link™ NHS-PEG4-Biotin kit (ThermoFisher). N00 was diluted to 5 µg/ml with octet buffer (20 mM sodium phosphate pH 7.5, 150 mM NaCl, 0.01% (w/v) LMNG) and loaded onto streptavidin (SA) biosensors (fortéBIO) that was hydrated with the same buffer. Unbound N00 was washed off with octet buffer. DtpA variants at 6.25 - 200 nM were then bound to N00. Experiments were performed at 25°C under shaking at 1000 rpm. Data were analyzed using Data Analysis v.10.0.3.1. software (fortéBIO) assuming a 1:1 stoichiometry of the DtpA-N00 complex. A Savitzky-Golay filter was applied to smooth the data.

Rotational isomeric state model for FRET efficiency prediction FRET prediction was done by first simulating possible dye positions $(\vec{s}_i, \vec{t}_j)$ along with their probabilities (p_i, q_j), $\{\vec{s}_i, p_i\}$ and $\{\vec{t}_j, q_j\}$, for donor and acceptor fluorophores. The FRET efficiency was then

evaluated as $E=\sum_i \sum_j \frac{R_0^6}{R_0^6+r_{ij}^6} p_i q_j$, where $r_{ij}=|\vec{s}_i-\vec{t}_j|$ and R_0=5.4 nm is the Förster radius. The values for $\{\vec{s}_i, p_i\}$ and $\{\vec{t}_j, q_j\}$ were obtained by modeling the fluorophores as bulky spheres, which were attached to the given C_β through a polyethylene chain of eleven monomers. The conformational freedom of the linker was accounted for using the rotational isomeric state model (RIS) [6]: each bond along the chain samples the anti, gauche+ and gauche- rotamers. Chains that sterically clashed [7] with the protein were discarded. For computational ease, the conformational space was sampled stochastically as a biased random walk and probabilities for each step were derived from the RIS. Convergence was reached after sampling 10,000 linker conformations (standard deviation in predicted FRET <0.003). Geometric and energetic parameters of the linker were taken from Rehan, M *et al.* [6], the fluorophore size from Kalinin, S. *et al.* [8], and the DtpA structure from PDB entry 6GS7.

Single-molecule FRET microscopy. Single-molecule FRET experiments were performed with a MicroTime 200 confocal system (PicoQuant, Germany) equipped with an Olympus IX73 inverted microscope and two pulsed excitation sources (40 MHz) controlled by a PDL 828-L "Sepia II" (PicoQuant, Germany). Pulsed Interleaved Excitation (PIE) [9–11] was used to identify molecules with active acceptor and donor dyes. Two light sources, a 485 nm Diode Laser (LDH-D-C-485, PicoQuant) and a white-light laser (Solea, PicoQuant) set to an excitation wavelength of 595 nm were used to excite the donor and the acceptor dyes alternatingly. The laser intensities were adjusted to 100 µW at 485 nm and 20 µW at 595 nm (Pm100D, Thor Labs). The excitation beams were guided through a major dichroic mirror (ZT 470-491/594 rpc, Chroma) to a 60x1.2 NA water objective (Olympus) where they were focused into the sample. The samples were placed on quartz and glass coverslips (Esco Optics) with a 6 mm diameter cloning cylinder (borosilicate glass, Hilgenberg) glued on top. The experiments were conducted in fresh buffers containing 20 mM sodium phosphate, pH 7.5, 150 mM NaCl, 0.002 % LMNG and 20 mM DTT. The experiments with reconstituted variants in SapNPs were performed in the same buffer without the LMNG component. We verified that the addition of DTT does not significantly affect the structural integrity of SapNPs and therefore the FRET histograms (SI Appendix, Fig. S18). The samples were diluted to 40-100 pM. Photons emitted from the sample were collected by the objective specified above. After passing the dichroic mirror (specified above), the residual excitation light was removed by a long-pass filter (BLP01-488R, Semrock) and the fluorescence light was focused on a 100 µm pinhole to remove out-of-focus light. Afterwards, the fluorescence photons were separated into donor and acceptor components using a dichroic mirror (585DCXR, Chroma, Rockingham, VT) and directed into donor and acceptor photon counting channels. Each light component was then focused onto the active area of a single-photon avalanche diode (SPAD) (Excelitas) after passing additional band-pass filters: FF03-525/50, (Semrock) for the donor and FF02-650/100 (Semrock) for the acceptor. For fluorescence anisotropy measurements, the emission light was first separated into its parallel and perpendicular components with respect to the linearly polarized excitation light via a polarizing beam splitter and each component was separated by two dichroic mirrors into donor and acceptor photons. The arrival time of the detected photons was recorded with a HydraHarp 400 time-correlated single photon counting (TCSPC) module (PicoQuant) and temperature controlled smFRET experiments were executed in a custom-made system (SI Appendix, Fig. S20), adapted from Nettels et al. and Aznauryan et al. [12–14]. The temperature inside the confocal spot was determined by precisely measuring the diffusion coefficient of Oregon Green (ThermoFisher Scientific) in water using 2f-FCS [15] (SI Appendix, Fig. S21). The average of the fits from three independent measurements at each temperature were used to determine the viscosity of water given the hydrodynamic radius of Oregon Green, (0.6 nm) [15,16]. The known temperature dependence of the water viscosity [17] was then used to calculate the temperature in the confocal spot. Errors in temperature (SI Appendix, Fig. S5-6, S22-24) are standard deviations of the triplicates.

SmFRET data analysis. As described previously [18,19], photons from individual molecules, separated by less than 100 µs were combined into bursts if the total number of photons exceeded 80. Photon counts were corrected for background, acceptor direct excitation, and different detection efficiencies of the individual detectors [20]. To remove potential aggregates, we only included bursts with a burst duration < 3 ms and a PIE stoichiometry ratio S < 0.7 [21]. FRET histograms were fitted with a combination of Gaussian and log-normal functions as previously described [22].

SmFRET histogram fitting. To obtain reliable fits of the transfer efficiency histograms, we first fitted the histograms of the reconstituted variants in various lipid composition SapNPs, supplemented with 8 µM N00 (Fig. 4B,D,E,F). These histograms were fitted with two Gaussian distributions where the peak position and width of molecules that lack an active acceptor was set to 0.00 and 0.06, respectively. The second population at intermediate FRET was then fitted with a Gaussian distribution. The resulting peak positions and widths were then used to fit the histograms in the absence of N00, which provided a reliable determination of the peaks at high (log-normal peak, inward closed) and very low (Gaussian peak, extremely inward open) FRET. To minimize the number of free fitting parameters, fits of the histograms in absence of N00 were performed globally for all four experimental conditions (Fig. 4B,D,E,F), with peak positions, widths, and asymmetries being global parameters. Since the high-FRET population is only marginally populated in LMNG, we used the position, width, and asymmetry resulting from the global fit of the data obtained in SapNPs also in fits of the data in LMNG. The dominant peak at intermediate FRET was then fitted without constraints.

Fluorescence lifetime analysis. The average lifetimes of the donor in the presence of acceptor τ_{DA} (Fig. 2A,C and S16) is estimated by the mean arrival time of the photons relative to the exciting laser pulse. The expected value of the fluorescence lifetime of the donor in the presence of the acceptor τ_{DA} for a static inter-dye distance is given by: $\tau_{DA}/\tau_D = 1 - E$, where τ_D = 4 ns is the lifetime of the donor in the absence of acceptor [23–25]. In contrast, a distribution of distances $P(r)$ alters this dependence [26]. To determine the width of this distribution, we fit the peak position of the average fluorescence lifetime $\langle\tau_{DA}\rangle$ and mean FRET efficiency $\langle E\rangle$ using the empirical distance distribution $P(r) = 4\pi r^2 exp[-(r-\mu)/2s^2]$ [27]. Notably, the parameters μ and s do not directly correspond to the position and width of the distance distribution. Instead, they are computed from the first and second moments of the distribution. The position is then

given by the first moment $\langle r\rangle = \int_0^\infty rP(r)dr \big/ \int_0^\infty P(r)dr$ and the width is given by $\sigma^2 = \langle r^2\rangle - \langle r\rangle^2$. The analytical expressions for the average lifetime and FRET efficiency are given by $\langle\tau_{DA}\rangle = \int_0^\infty \tau_{DA}(r)^2 P(r)dr \big/ \int_0^\infty \tau_{DA}(r)P(r)dr$ and $\langle E\rangle = \int_0^\infty E(r)P(r)dr \big/ \int_0^\infty P(r)dr$, respectively. Here, $\tau_{DA}(r) = \tau_D[1 + (R_0/r)^6]^{-1}$ and $E(r) = R_0^6/(R_0^6 + r^6)$ with R_0 being the Förster distance of our dye pair [26] (5.4 nm).

Recurrence analysis of single particles (RASP). The recurrence analysis of single particles (RASP) uses the fact that a freely diffusing molecule can be observed multiple times in a single-molecule experiment (Fig. 3A, main text). Once a molecule leaves the observation volume, the chance to return to the confocal spot is greater than the chance of detecting a new molecule for short time intervals. This effect can be used to extract the kinetics of forming the low-FRET state from molecules in the high-FRET state and *vice versa*. To extract the kinetics, we first identify all bins with transfer efficiencies indicative of the high-FRET state ($0.7 < E \geq 1.2$). In a second step, we construct FRET-histograms for those bins that follow the originally identified set in the window $(\tau - T, \tau)$, with τ being the time shift and T being the window size. With increasing τ, the relative population of high-FRET molecules decays in the recurrence histograms while the population of low-FRET molecules increases (Fig. 3B). Two factors contribute to this redistribution: (*i*) the conformational dynamics of DtpA, i.e., molecules that were initially in the high-FRET state switch to the low-FRET state at longer times, and (*ii*) the arrival of new molecules in the observation volume. To disentangle the two contributions, we determine $p_{same}(\tau)$, which is the probability that two bins at time t_1 and t_2 (with $\tau = t_2 - t_1$) result from the same molecule. This quantity is directly accessible from the bin-pair autocorrelation functions [28] and characterizes the frequency with which new molecules enter the confocal volume. The bin-pair autocorrelation function is defined as

$$g(\tau) = p(\{b_1, t\}, \{b_2, t + \tau\}) / p(\{b_1, t\}) p(\{b_2, t\}) \qquad (1)$$

Here, $p(\{b_1, t\}, \{b_2, t + \tau\})$ denotes the joint probability of observing two bins b_1 and b_2 at times t and $t + \tau$, respectively, and $p(\{b_1, t\})$ and $p(\{b_2, t\})$ are the probabilities of detecting bin b_1 and b_2, respectively, at time t. Thus, eq. 1 provides the timescale up to which the occurrence of successive bins is correlated and it directly relates to the probability that two successive bins b_1 and b_2 are from the same molecule, which is given by

$$p_{same}(\tau) = 1 - 1/g(\tau). \qquad (2)$$

The knowledge of $p_{same}(\tau)$ can now be used to compute the RASP-kinetics in the absence of conformational exchange, i.e., for heterogeneity that is static on the recurrence timescale of 100 μs – 10 ms in our case. The observed increase in the population of low-FRET molecules in the recurrence histograms after selecting high-FRET molecules initially, $p_{obs}(\tau)$, is then given by

$$p_{obs}(\tau) = p_{same}(\tau) p_{conf}(\tau) + [1 - p_{same}(\tau)]\rho_{eq} \qquad (3)$$

Here, $p_{conf}(\tau)$ is the kinetics of forming the open state due to conformational dynamics and ρ_{eq} is the equilibrium fraction of low-FRET molecules, which is accessible from the measured FRET-histogram (Fig. 2A,B). In the absence of conformational dynamics, i.e., for $p_{conf}(\tau) \to 0$, eq. 3 reduces to

$$p_{obs}(\tau) = [1 - p_{same}(\tau)]\rho_{eq} \qquad (4)$$

which provides the kinetics in the sole presence of static heterogeneity. In the presence of conformational exchange however, the term $p_{conf}(\tau)$ is the time-dependent fraction of low-FRET molecules that arise due to conformational transitions from the closed state. The recurrence FRET-histograms at different delay time were globally fitted with a sum of a log-normal function for the high-FRET state, a Gaussian function for the population of the low-FRET molecules, and a Gaussian peak for molecules that lack an active acceptor dye (E = -0.05). Here, the position of the high- and low-FRET populations were fixed to the positions obtained in the equilibrium histograms, while widths, asymmetries, and amplitudes were free fitting parameters. The relative populations of low-FRET and high-FRET molecules were obtained by integrating the respective sub-populations. After determining the conformational switching kinetics $p_{conf}(\tau)$ using eq. 3, we used a single-exponential fit to determine the rate of switching.

Sequence information

Wildtype DtpA sequence

ATGTCCACTGCAAACCAAAAACCAACTGAAAGCGTCAGTTTGAACGCTTTCAAACAACCGAAGGCGTTCTATCTCATCTTCTCGAT
TGAGTTATGGGAACGTTTTGGTTATTACGGCCTACAAGGAATTATGGCTGTTTACCTGGTTAAACAACTGGGTATGTCTGAAGCG
GATTCAATCACCCTTTTCTCTTCCTTTAGTGCCCTGGTTTATGGTCTGGTCGCTATCGGCGGCTGGTTAGGTGACAAGGTACTGG
GTACTAAACGCGTAATTATGCTCGGCGCTATTGTGCTGGCGATTGGTTATGCTCTGGTTGCCTGGTCTGGTCACGACGCCGGTA
TCGTTTATATGGGTATGGCGGCTATTGCGGTCGGTAACGGCCTGTTTAAAGCTAACCCGTCTTCTCTGCTTTCTACATGCTATGA
GAAAAACGACCCGCGTCTGGACGGTGCATTCACCATGTACTACATGTCCGTCAACATCGGCTCTTTCTTCTCTATGATTGCTACG
CCGTGGCTGGCCGCGAAATACGGCTGGAGTGTTGCGTTTGCGTTGAGCGTTGTAGGCCTGCTGATCACTATCGTTAACTTCGCC
TTCTGCCAACGCTGGGTTAAACAGTACGGTTCAAAACCAGACTTCGAGCCTATCAACTACCGTAACCTGCTGCTGACCATTATTG
GTGTTGTGGCACTGATCGCTATCGCCACCTGGCTGCTGCACAATCAGGAAGTTGCGCGTATGGCGCTGGGCGTTGTTGCCTTC
GGTATCGTGGTTATCTTCGGTAAAGAAGCCTTCGCGATGAAAGGTGCTGCGCGTCGTAAAATGATCGTTGCCTTCATCCTGATGC

TCGAAGCCATTATCTTCTTCGTGCTGTACAGCCAGATGCCAACGTCACTGAACTTCTTTGCGATTCGTAACGTTGAGCACTCCATT
CTGGGTCTGGCCGTAGAACCTGAGCAGTATCAGGCACTGAACCCGTTCTGGATCATCATCGGTAGTCCGATTCTGGCCGCTATC
TATAACAAGATGGGCGATACCCTGCCGATGCCAACCAAGTTTGCAATCGGCATGGTGATGTGTTCTGGTGCGTTCCTGATTCTGC
CGCTGGGTGCGAAATTCGCGTCTGACGCTGGTATCGTGTCTGTAAGCTGGCTGGTCGCAAGCTATGGCCTGCAGAGCATCGGG
GAACTGATGATCTCTGGTCTGGGTCTGGCAATGGTTGCTCAACTCGTTCCGCAGCGTCTGATGGGCTTCATTATGGGTAGCTGG
TTCCTGACCACTGCCGGTGCAAACCTGATTGGTGGTTATGTTGCGGGTATGATGGCTGTGCCGGATAACGTTACCGATCCGCTG
ATGTCACTGGAAGTCTATGGTCGCGTATTCTTGCAGATTGGTGTCGCTACTGCCGTTATTGCAGTACTGATGCTGCTGACCGCGC
CGAAACTGCACCGCATGACGCAGGATGACGCTGCAGACAAAGCGGCGAAAGCAGCCGTAGCGGCAGAGAACCTCTACTTCCAA
TCGCACCATCATCACCACCATGATTACAAGGATGACGACGATAAGTGA

W203C/Q487C variant sequence

ATGTCCACTGCAAACCAAAAACCAACTGAAAGCGTCAGTTTGAACGCTTTCAAACAACCGAAGGCGTTCTATCTCATCTTCTCGAT
TGAGTTATGGGAACGTTTTGGTTATTACGGCCTACAAGGAATTATGGCTGTTTACCTGGTTAAACAACTGGGTATGTCTGAAGCG
GATTCAATCACCCTTTTCTCTTCCTTTAGTGCCCTGGTTTATGGTCTGGTCGCTATCGGCGGCTGGTTAGGTGACAAGGTACTGG
GTACTAAACGCGTAATTATGCTCGGCGCTATTGTGCTGGCGATTGGTTATGCTCTGGTTGCCTGGTCTGGTCACGACGCCGGTA
TCGTTTATATGGGTATGGCGGCTATTGCGGTCGGTAACGGCCTGTTTAAAGCTAACCCGTCTTCTCTGCTTTCTACATCGTATGA
GAAAAACGACCCGCGTCTGGACGGTGCATTCACCATGTACTACATGTCCGTCAACATCGGCTCTTTCTTCTCTATGATTGCTACG
CCGTGGCTGGCCGCGAAATACGGCTGGAGTGTTGCGTTTGCGTTGAGCGTTGTAGGCCTGCTGATCACTATCGTTAACTTCGCC
TTCTCACAACGCTGCGTTAAACAGTACGGTTCAAAACCAGACTTCGAGCCTATCAACTACCGTAACCTGCTGCTGACCATTATTG
GTGTTGTGGCACTGATCGCTATCGCCACCTGGCTGCTGCACAATCAGGAAGTTGCGCGTATGGCGCTGGGCGTTGTTGCCTTC
GGTATCGTGGTTATCTTCGGTAAAGAAGCCTTCGCGATGAAAGGTGCTGCGCGTCGTAAAATGATCGTTGCCTTCATCCTGATGC
TCGAAGCCATTATCTTCTTCGTGCTGTACAGCCAGATGCCAACGTCACTGAACTTCTTTGCGATTCGTAACGTTGAGCACTCCATT
CTGGGTCTGGCCGTAGAACCTGAGCAGTATCAGGCACTGAACCCGTTCTGGATCATCATCGGTAGTCCGATTCTGGCCGCTATC
TATAACAAGATGGGCGATACCCTGCCGATGCCAACCAAGTTTGCAATCGGCATGGTGATGTCATCTGGTGCGTTCCTGATTCTGC
CGCTGGGTGCGAAATTCGCGTCTGACGCTGGTATCGTGTCTGTAAGCTGGCTGGTCGCAAGCTATGGCCTGCAGAGCATCGGG
GAACTGATGATCTCTGGTCTGGGTCTGGCAATGGTTGCTCAACTCGTTCCGCAGCGTCTGATGGGCTTCATTATGGGTAGCTGG
TTCCTGACCACTGCCGGTGCAAACCTGATTGGTGGTTATGTTGCGGGTATGATGGCTGTGCCGGATAACGTTACCGATCCGCTG
ATGTCACTGGAAGTCTATGGTCGCGTATTCTTGCAGATTGGTGTCGCTACTGCCGTTATTGCAGTACTGATGCTGCTGACCGCGC
CGAAACTGCACCGCATGACGTGCGATGACGCTGCAGACAAAGCGGCGAAAGCAGCCGTAGCGGCAGAGAACCTCTACTTCCAA
TCGCACCATCATCACCACCATGATTACAAGGATGACGACGATAAGTGA

W203C/T351C variant sequence

ATGTCCACTGCAAACCAAAAACCAACTGAAAGCGTCAGTTTGAACGCTTTCAAACAACCGAAGGCGTTCTATCTCATCTTCTCGAT
TGAGTTATGGGAACGTTTTGGTTATTACGGCCTACAAGGAATTATGGCTGTTTACCTGGTTAAACAACTGGGTATGTCTGAAGCG
GATTCAATCACCCTTTTCTCTTCCTTTAGTGCCCTGGTTTATGGTCTGGTCGCTATCGGCGGCTGGTTAGGTGACAAGGTACTGG
GTACTAAACGCGTAATTATGCTCGGCGCTATTGTGCTGGCGATTGGTTATGCTCTGGTTGCCTGGTCTGGTCACGACGCCGGTA
TCGTTTATATGGGTATGGCGGCTATTGCGGTCGGTAACGGCCTGTTTAAAGCTAACCCGTCTTCTCTGCTTTCTACATCGTATGA
GAAAAACGACCCGCGTCTGGACGGTGCATTCACCATGTACTACATGTCCGTCAACATCGGCTCTTTCTTCTCTATGATTGCTACG
CCGTGGCTGGCCGCGAAATACGGCTGGAGTGTTGCGTTTGCGTTGAGCGTTGTAGGCCTGCTGATCACTATCGTTAACTTCGCC
TTCTCACAACGCTGCGTTAAACAGTACGGTTCAAAACCAGACTTCGAGCCTATCAACTACCGTAACCTGCTGCTGACCATTATTG
GTGTTGTGGCACTGATCGCTATCGCCACCTGGCTGCTGCACAATCAGGAAGTTGCGCGTATGGCGCTGGGCGTTGTTGCCTTC
GGTATCGTGGTTATCTTCGGTAAAGAAGCCTTCGCGATGAAAGGTGCTGCGCGTCGTAAAATGATCGTTGCCTTCATCCTGATGC
TCGAAGCCATTATCTTCTTCGTGCTGTACAGCCAGATGCCAACGTCACTGAACTTCTTTGCGATTCGTAACGTTGAGCACTCCATT
CTGGGTCTGGCCGTAGAACCTGAGCAGTATCAGGCACTGAACCCGTTCTGGATCATCATCGGTAGTCCGATTCTGGCCGCTATC
TATAACAAGATGGGCGATACCCTGCCGATGCCATGTAAGTTTGCAATCGGCATGGTGATGTCATCTGGTGCGTTCCTGATTCTGC
CGCTGGGTGCGAAATTCGCGTCTGACGCTGGTATCGTGTCTGTAAGCTGGCTGGTCGCAAGCTATGGCCTGCAGAGCATCGGG
GAACTGATGATCTCTGGTCTGGGTCTGGCAATGGTTGCTCAACTCGTTCCGCAGCGTCTGATGGGCTTCATTATGGGTAGCTGG
TTCCTGACCACTGCCGGTGCAAACCTGATTGGTGGTTATGTTGCGGGTATGATGGCTGTGCCGGATAACGTTACCGATCCGCTG
ATGTCACTGGAAGTCTATGGTCGCGTATTCTTGCAGATTGGTGTCGCTACTGCCGTTATTGCAGTACTGATGCTGCTGACCGCGC
CGAAACTGCACCGCATGACGCAGGATGACGCTGCAGACAAAGCGGCGAAAGCAGCCGTAGCGGCAGAGAACCTCTACTTCCAA
TCGCACCATCATCACCACCATGATTACAAGGATGACGACGATAAGTGA

Nanobody sequence

ATGGCCCAGGTGCAGCTGCAGGAGTCTGGAGGAGGATTGGTGCAGGCTGGGGGCTCTCTGAGACTCTCCTGTGCAGGCTCTG
GCCGCACCTTCAGTAGTTATAACATGGGCTGGTTCCGGCAGGCTCCAGGGAAGGAGCGTGAGTTTGTAGGAGGTATTAGCTGG
ACTGGTCGTAGTGCCGACTATCCAGACTCCGTGAAGGGCCGATTCACTATCTCCAGAGACAACGCCAAGAACGCGGTGTATCTG
CAAATGAACAGCCTGAAACCTGAAGACACGGCCGTTTATTACTGTGCAGCAAAGCAATACGGTAGTCGTGCTGACTACCCTTGG
GATGACTATGACTACTGGGGCCAGGGGACCCAGGTCACCGTCTCCTCAGGGGCAGCGGAACCTGAAGCCTAG

Saposin A sequence

ATGCACCATCATCATCATCATTCTTCTGGTGTAGATCTGGGTACCGAGAACCTGTACTTCCAATCCATGGGATCCCTTCCCTGCG
ACATATGCAAAGACGTTGTCACCGCAGCTGGTGATATGCTGAAGGACAATGCCACTGAGGAGGAGATCCTTGTTTACTTGGAGA
AGACCTGTGACTGGCTTCCGAAACCGAACATGTCTGCTTCATGCAAGGAGATAGTGGACTCCTACCTCCCTGTCATCCTGGACAT
CATTAAAGGAGAAATGAGCCGTCCTGGGGAGGTGTGCTCTGCTCTCAACCTCTGCGAGTCTTGA.

Table S1. Primer design outline for point mutations in wildtype DtpA.

Mutation	Forward primer (5'-3')	Reverse primer (5'-3')
C140S	GATGTAGAAAGCAGAGAAGACG	GTATGAGAAAAACGACCCG
C200S	GAGAAGGCGAAGTTAACGATA	ACAACGCTGGGTTAAACAGTA
W203C	CAGCGTTGTGAGAAGG	CGTTAAACAGTACGGTTCAA
T351C	ATGGCATCGGCAGGGTAT	GTAAGTTTGCAATCGGCATG
C360S	GACATCACCATGCCGATTGC	ATCTGGTGCGTTCCTGATTCT
Q487C	CACGTCATGCGGTGCAG	CGATGACGCTGCAGACAAA

Results

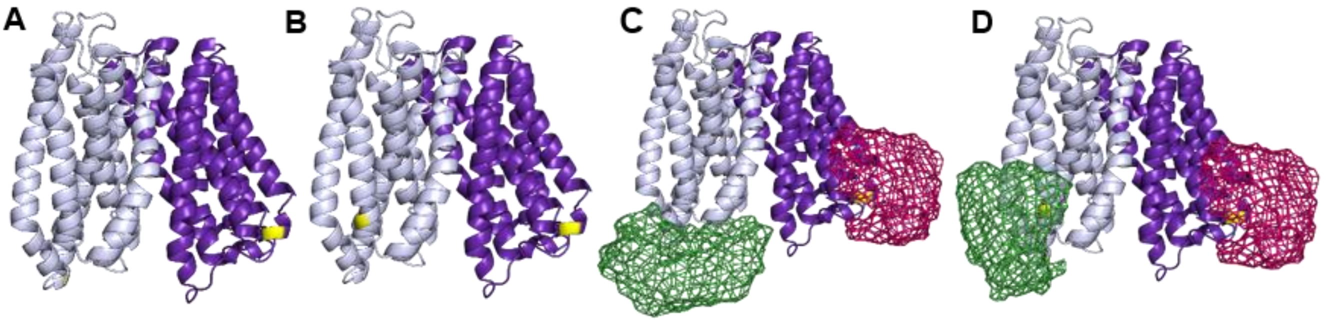

Fig. S1. **Labeling positions of DtpA variants. (A)** Labeling positions (yellow) of DtpA variant WQ (W203C/ Q487C) and **(B)** variant WT (W203C/ T351C). The N-terminal domain is colored purple and the C- terminal domain is shown in white. **(C)** WQ and **(D)** WT variants with FRET dye positional distribution (mesh) simulated with the Rotational Isomeric State Model. The simulation predicted mean FRET efficiency of 0.54 and 0.47 for WQ and WT respectively.

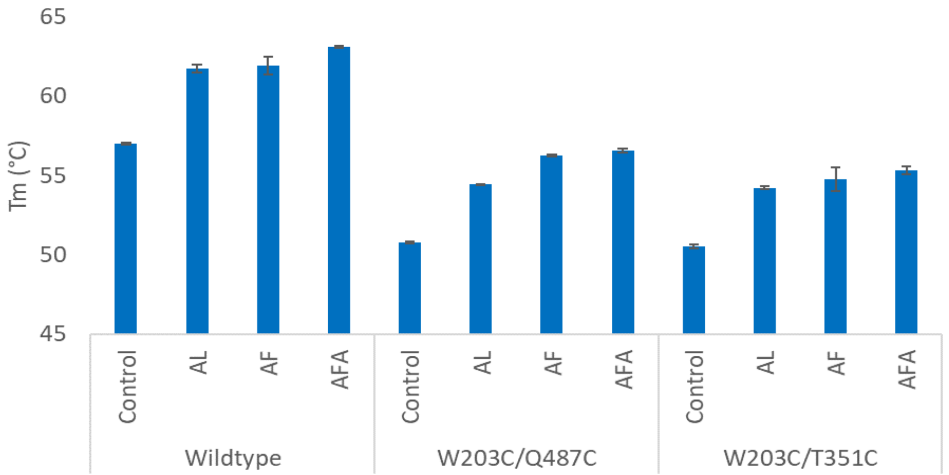

Fig. S2. Ligand binding of DtpA variants. Thermal stability determined by nanoDSF of the DtpA variants WQ and WT, and the wildtype in the presence of the ligands Ala-Leu (AL), Ala-Phe (AF) and Ala-Phe-Ala (AFA). Error bars represent standard deviations of triplicates.

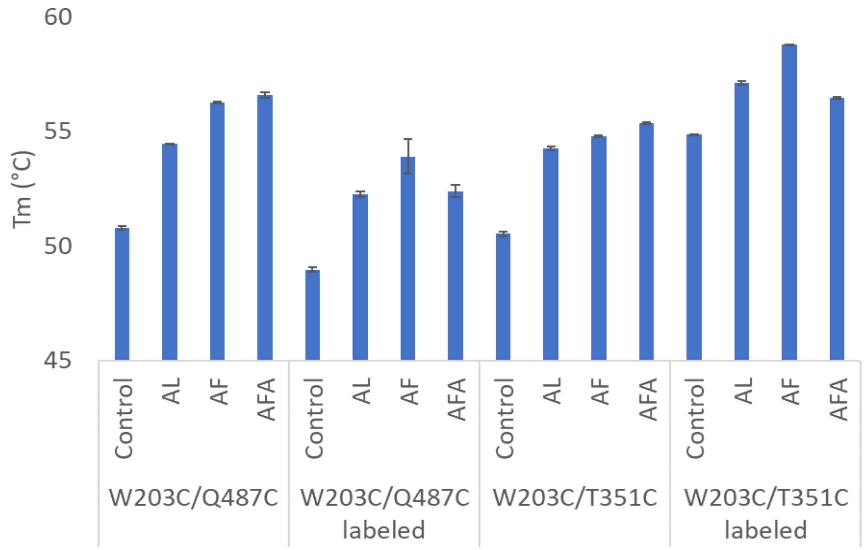

Fig. S3. Ligand binding of labeled DtpA variants. Thermal stability measured by nanoDSF of labeled and unlabeled variants of WQ and WT in the presence of the ligands Ala-Leu (AL), Ala-Phe (AF) and Ala-Phe-Ala (AFA). Error bars represent the standard deviation of triplicates.

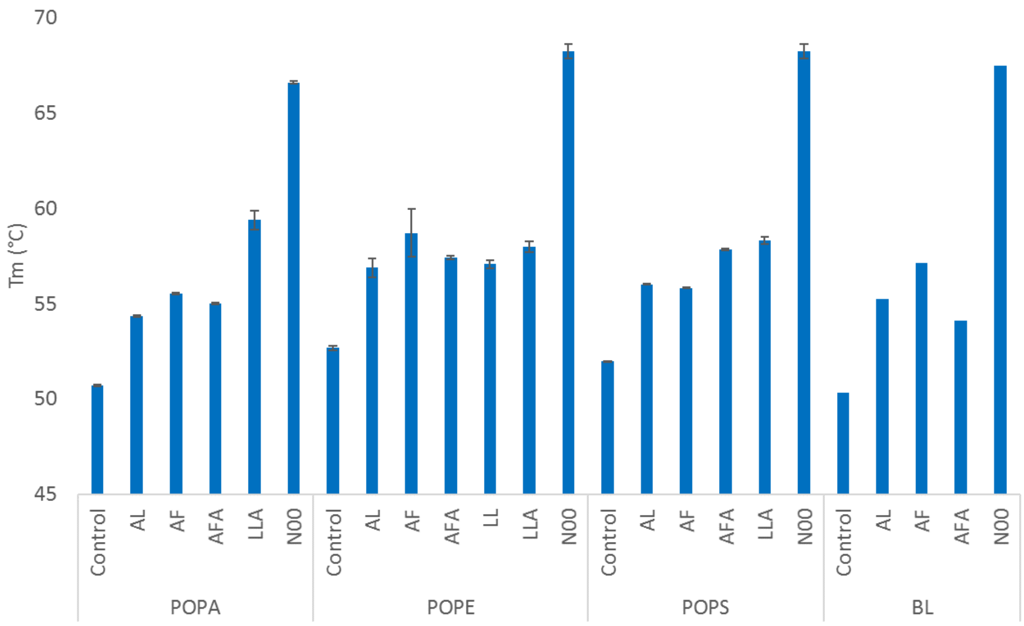

Fig. S4. Ligand binding of WQ in SapNPs. Thermal stability measured by nanoDSF of WQ in SapNPs of various lipid compositions: POPA, POPE, POPS and brain-lipids extract (BL) in the presence of ligands and N00. Error bars represent standard deviations of triplicates.

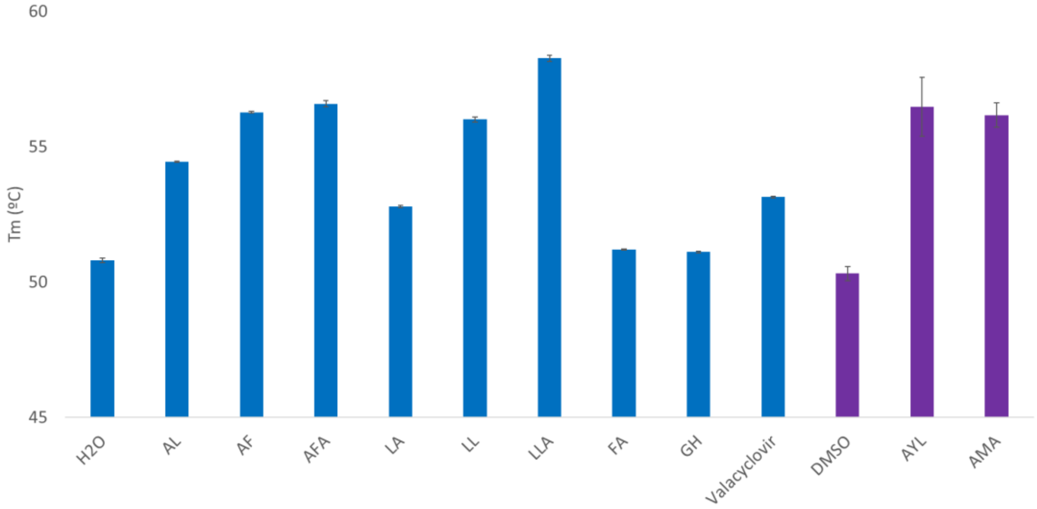

Fig. S5. Ligand binding of WQ. Thermal stability measured by nanoDSF of WQ in the presence of 2.5 mM of the following ligands: Ala-Leu (AL), Ala-Phe (AF), Ala-Phe-Ala (AFA), Leu-Ala (LA), Leu-Leu (LL), Phe-Ala (FA), Gly-His (GH), valacyclovir, Ala-Tyr-Leu (AYL) and Ala-Met-Ala (AMA). AL, AF, AFA, LA, LL, FA, GH and valacyclovir were pre-dissolved in water, AYL and AMA in 100% DMSO. Error bars represent standard deviations of triplicates.

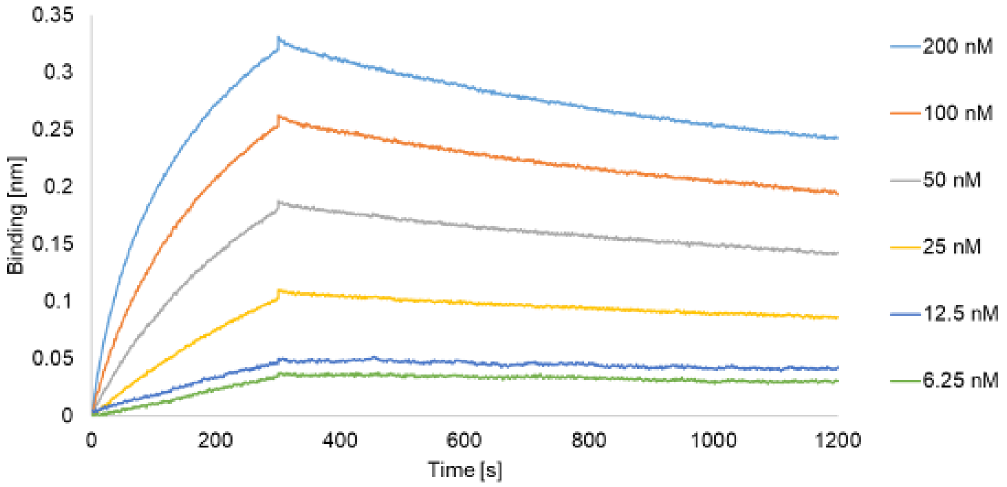

Fig. S6. Binding affinity of wildtype DtpA to nanobody N00. Binding affinity of wildtype DtpA to surface immobilized N00 determined by biolayer interferometry. The concentration of DtpA is indicated on the right.

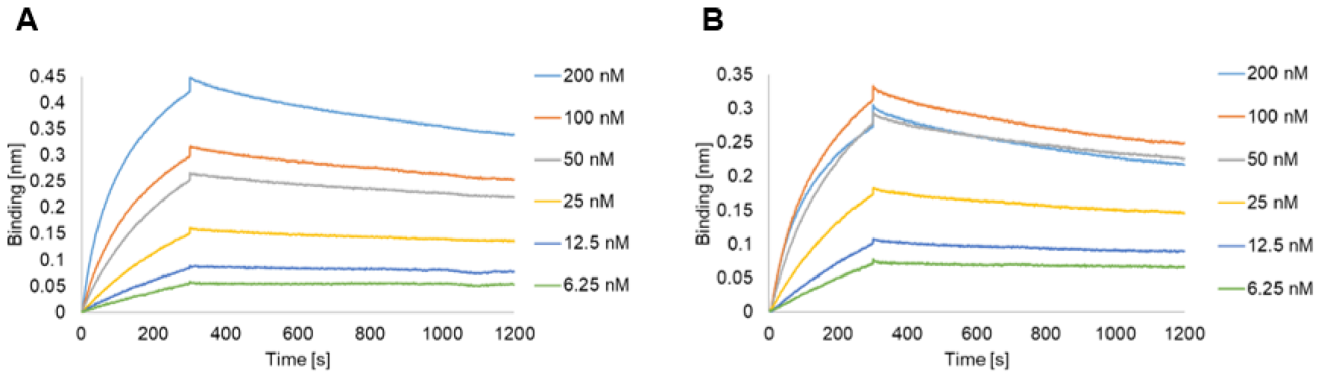

Fig. S7. Binding affinity of labeled DtpA variants to nanobody N00. (A) WQ_{DA} and **(B)** WT_{DA} variants to surface immobilized N00 determined by biolayer interferometry. The concentration of DtpA is indicated on the right of each panel.

Table S2. K_D values of N00 binding to DtpA variants. K_D values for wildtype DtpA, labeled WQ and WT, and the unlabeled WQ and WT variants were determined by the ratio of the dissociation to association rate as obtained by biolayer interferometry (Octet). Biotinylated N00 was loaded on Streptavidin biosensors and the binding was assessed at 200 nM, 100 nM, 50 nM, 25 nM, 12.5 nM and 6.25 nM DtpA and its variants. The analysis was performed using the Data Analysis software v.10.0.3.1 (fortéBIO) assuming a 1:1 stoichiometry of the protein-N00 complex.

Sample	K_D
Wildtype	7.13 ± 0.03 nM
W203C/T351C labeled	6.31 ± 0.05 nM
W203C/Q487C	6.28 ± 0.03 nM
W203C/Q487C labeled	5.40 ± 0.03 nM

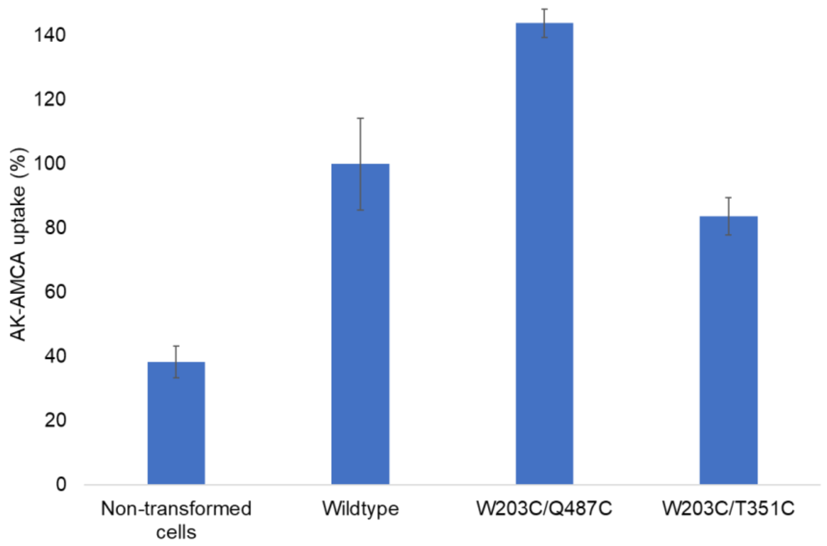

Fig. S8. Uptake assay of DtpA variants. *In vivo* uptake assay of AK-AMCA for non-transformed cells, wildtype DtpA and the variants WQ and WT. Error bars represent the standard deviation of triplicates.

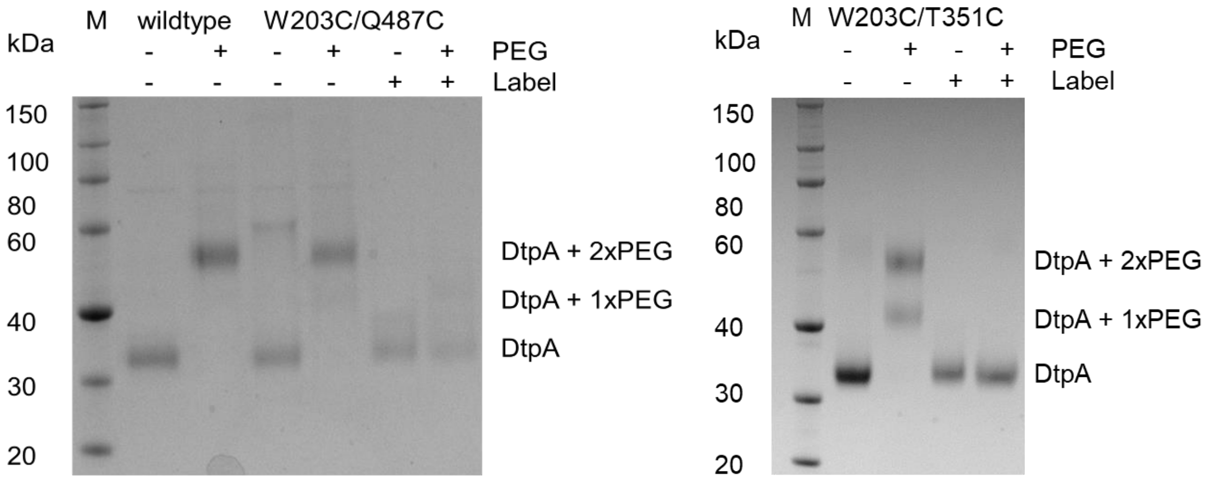

Fig. S9. Cysteine accessibility test - PEGylation assay of DtpA wildtype and variants WQ and WT. Labeled ("+" with Alexa Fluor maleimide) and unlabeled ("-") proteins were incubated with PEG-maleimide to visualize the accessibility of the cysteine residues. The attachment of PEG-maleimide to the free cysteine residues leads to a shift of the protein bands on SDS-PAGE to higher molecular weight (interpretation on the right). Thus, the WQ and WT cysteines were accessible for PEG-maleimide labeling via two sites, and were not accessible after labeling with the fluorescent dyes.

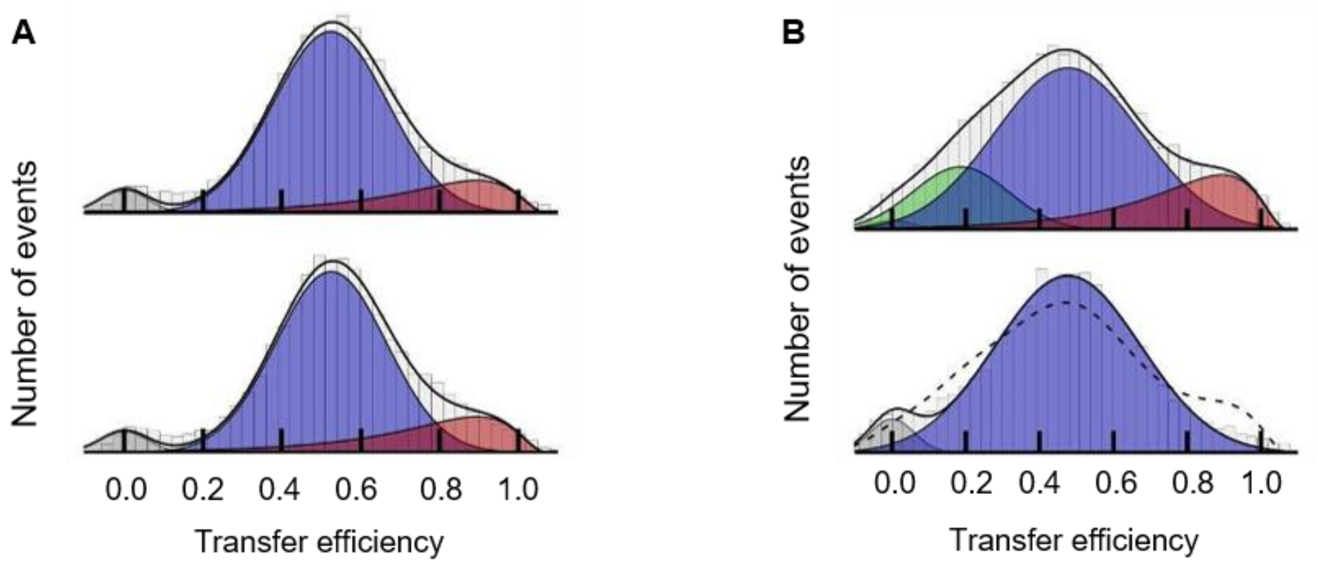

Fig. S10. SmFRET of WT. (A) SmFRET histograms of the WT_{DA} variant in LMNG without (top) and with (bottom) 8 µM N00 and **(B)** WT_{DA} reconstituted in POPS SapNPs without (top) and with 8 µM N00 (bottom). Solid lines are fits with a superposition of Gaussian and log-normal functions.

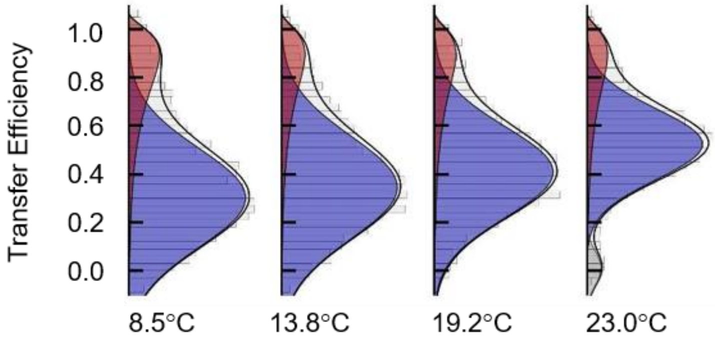

Fig. S11. SmFRET of WT at different temperature. SmFRET histograms of WT_{DA} variant in LMNG, as a function of temperature (indicated). Solid lines are fits with a superposition of Gaussian and log-normal functions.

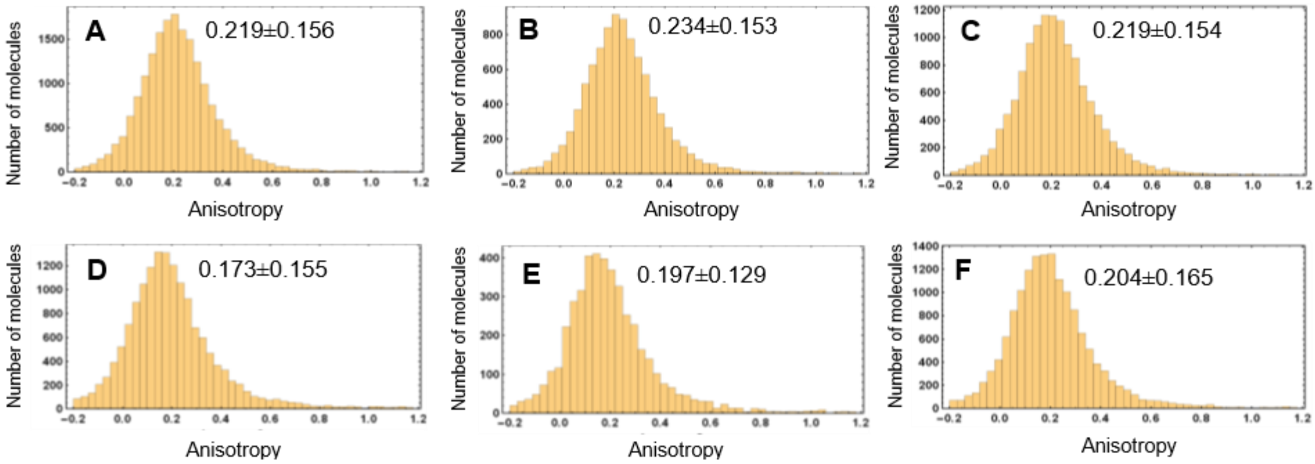

Fig. S12. FRET anisotropy of the labeled species (excluding molecules with inactive acceptor). (A) WQ_{DA} in LMNG, **(B)** WT_{DA} in LMNG, **(C)** WQ_{DA} in LMNG supplied with N00, **(D)** WQ_{DA} in POPE SapNPs, **(E)** WQ_{DA} in POPS SapNPs, **(F)** WQ_{DA} in POPE SapNPs supplied with N00.

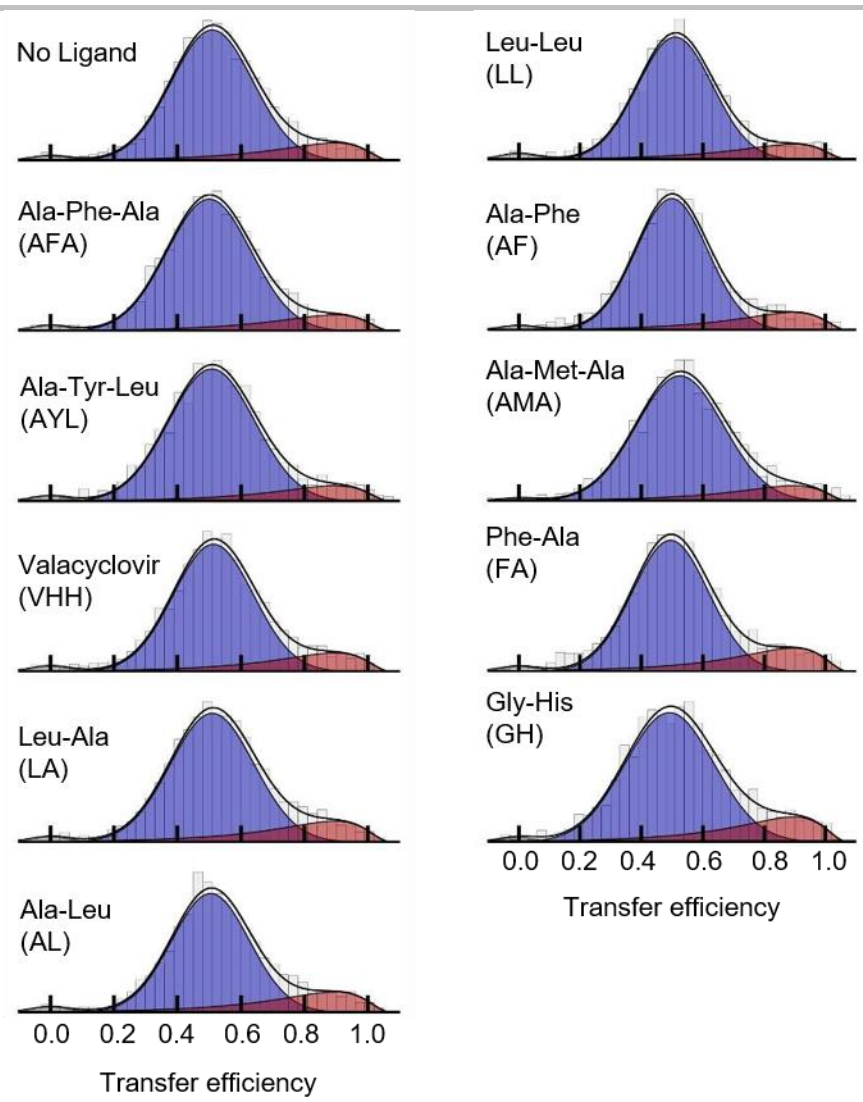

Fig. S13. SmFRET of WQ in the presence of ligands. SmFRET histograms of WQ_{DA} in LMNG with 2 mM ligands (indicated). Solid lines are fits with a superposition of Gaussian and log-normal functions.

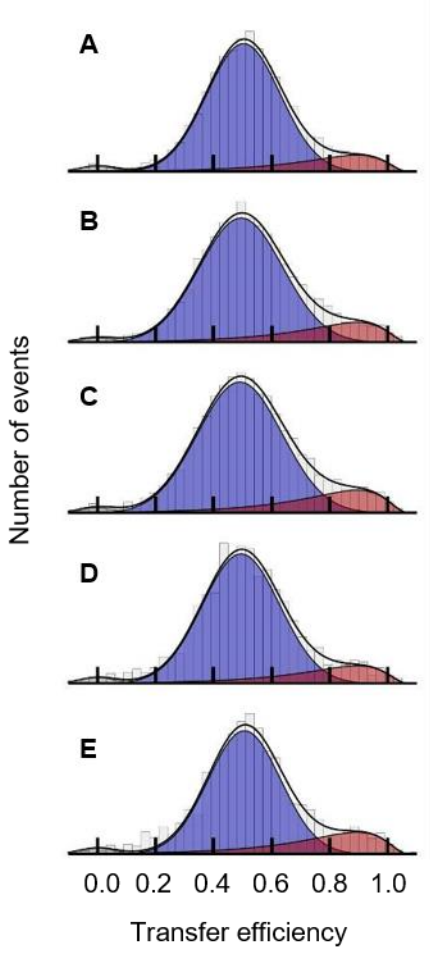

Fig. S14. SmFRET of WQ at different pH. (A) SmFRET histograms of WQ_{DA} in LMNG: pH = 7.5, **(B)** pH = 5.3, **(C)** pH = 5.3 supplied with 2 mM LL, **(D)** pH = 9.2, **(E)** pH = 9.2 supplied with 2 mM LL. Solid lines are fits with a superposition of Gaussian and log-normal functions.

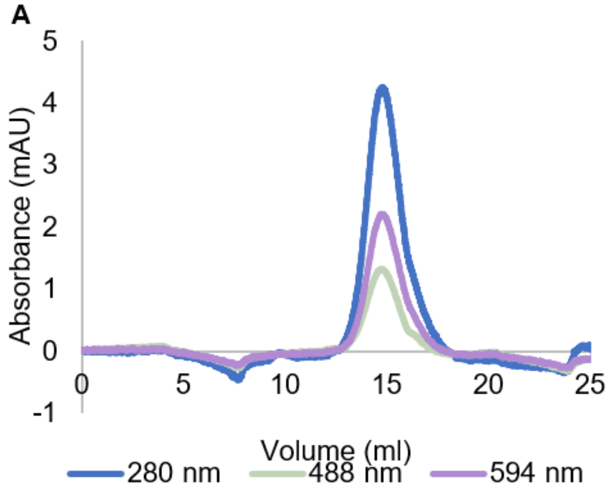

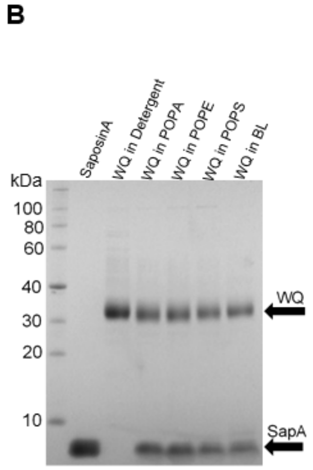

Fig. S15. Reconstitution of WQ into SapNPs. (A) Elution profile (SEC) of WQ_{DA} reconstituted into POPS SapNPs, run on a Superdex 200 Increase 10/300 GL column (GE Healthcare Life Sciences), confirming the absence of oligomeric or aggregated species. **(B)** SDS-PAGE of WQ before and after reconstitution into Sap-NPs with various lipids confirming SapNPs assembly by coelution of Saposin A and reconstituted WQ.

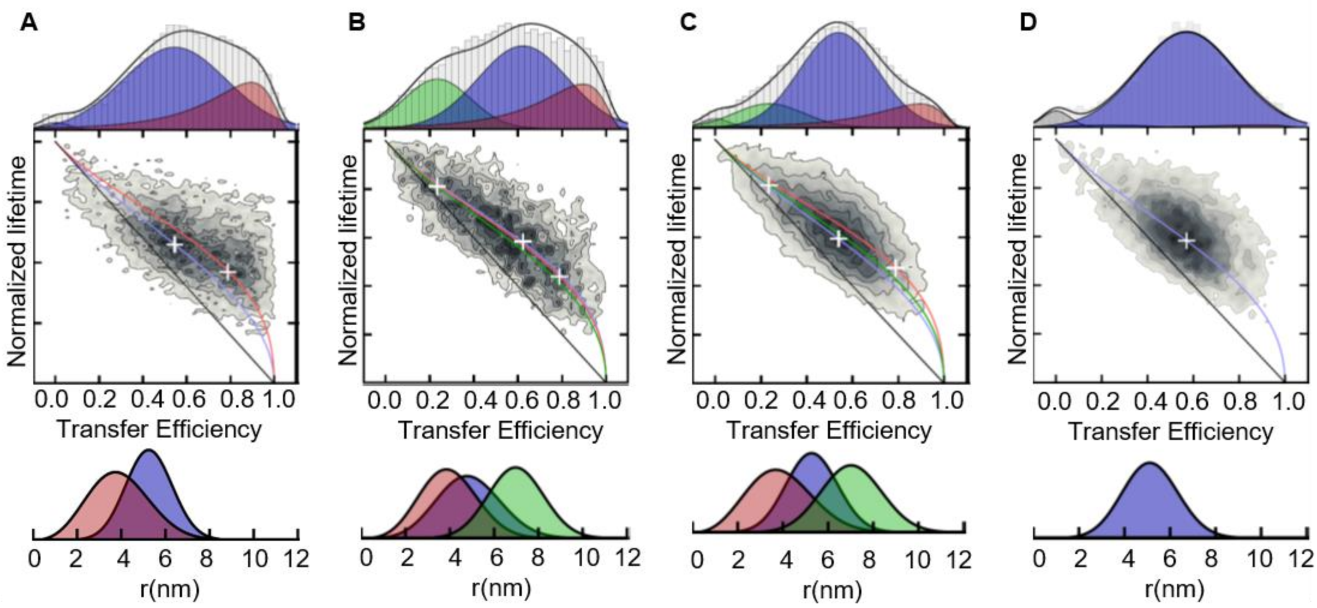

Fig. S16. SmFRET of WQ in SapNPs. SmFRET histograms (*top*) of DtpA WQ_{DA} in SapNPs composed of: **(A)** POPE, **(B)** POPS, **(C)** POPA and **(D)** BL. Solid lines are fits with Gaussian and log-normal peaks. 2D correlation map (*middle*) between donor fluorescence lifetime and transfer efficiency. Solid lines show the dependence for a single static donor-acceptor distance (black) and the best fit of the mean position of open, extreme open and closed molecules (white crosses) with a distance distribution (according to the population color). The distance distribution obtained from the fit *(bottom)*.

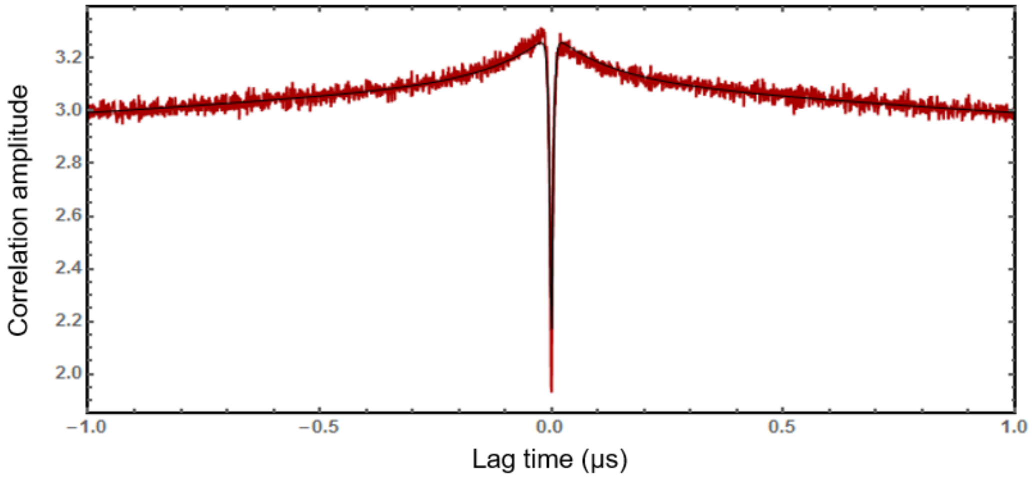

Fig. S17. Nanosecond FCS (nsFCS) of the acceptor signal of WQ. Measured of WQ_{DA} in POPS SapNPs after excitation at 594 nm (30 µW). Solid line is a fit including components for anti-bunching (3.8 ns), the correlated decay due to quenching (127 ns, amplitude 8.1%), and triplet blinking of the dye (3.5 s).

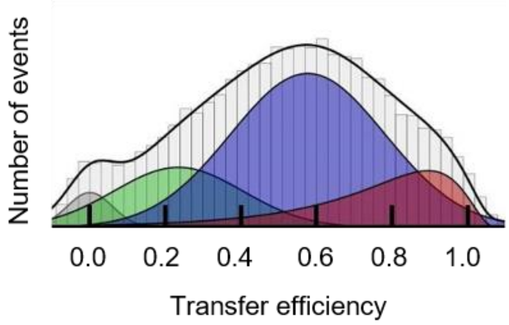

Fig. S18. SmFRET histograms of WQ_{DA} in POPS SapNPs in the absence of DTT. Solid lines are fits with a superposition of Gaussian and log-normal peaks.

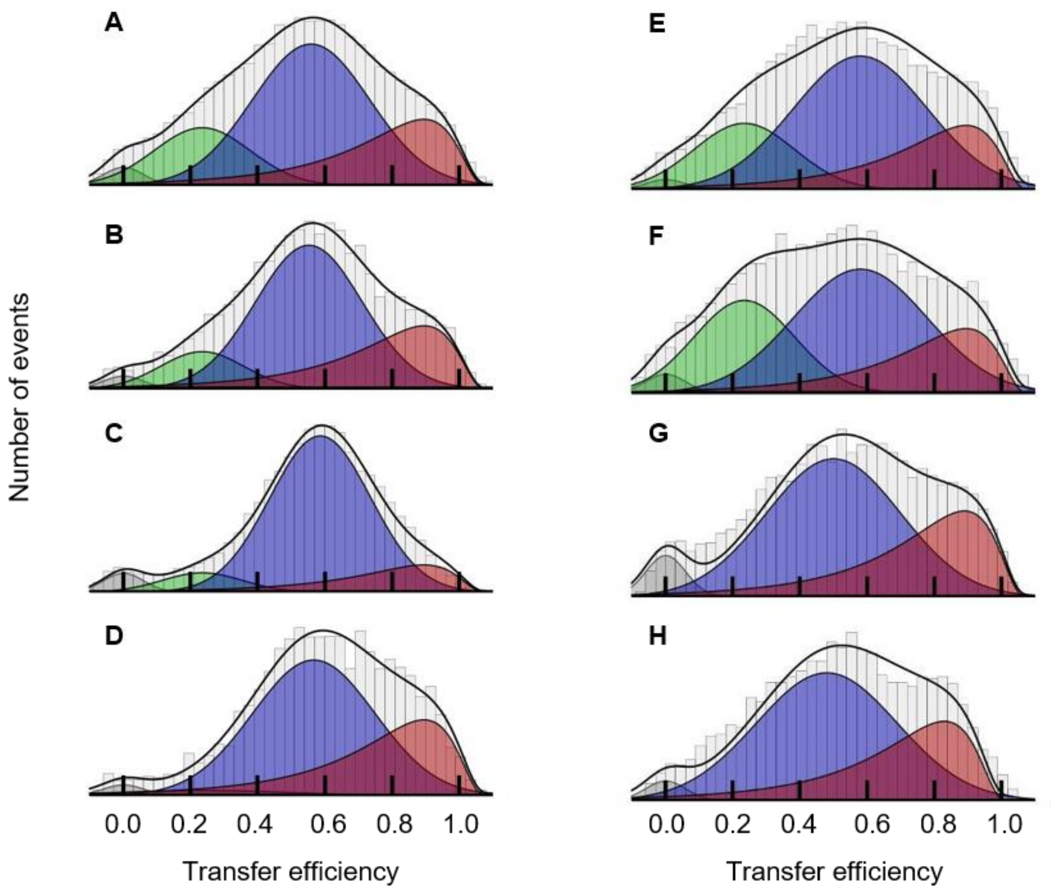

Fig. S19. SmFRET of WQ in SapNPs at different pH with and without ligand addition. SmFRET histograms of WQ_{DA} reconstituted in SapNPs **(A)** POPS supplied with 2 mM AF, **(B)** POPS supplied with 2 mM LLA, **(C)** POPA supplied with 2 mM LLA, **(D)** POPE supplied with 2 mM LLA, **(E)** POPS pH = 6.4, **(F)** POPS pH = 8.1, **(G)** POPE pH = 5.3 and **(H)** POPE pH = 5.3 supplied with 2 mM LL. Solid lines are fits with a superposition of Gaussian and log-normal functions.

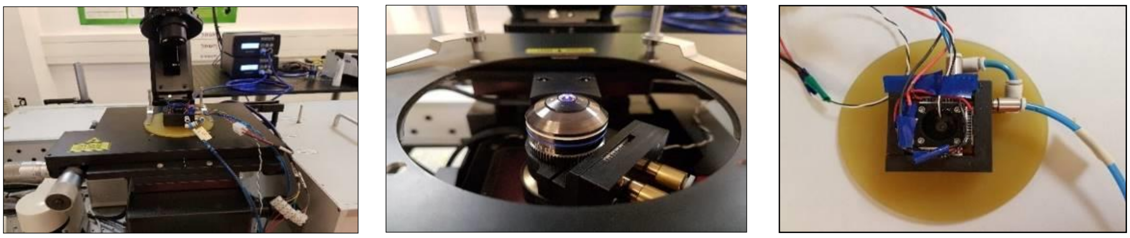

Fig. S20. Temperature-controlled sample holder for single-molecule smFRET experiments. The cell adjusted over the objective (left). The objective sleeve (center), cell with a cuvette top view (right).

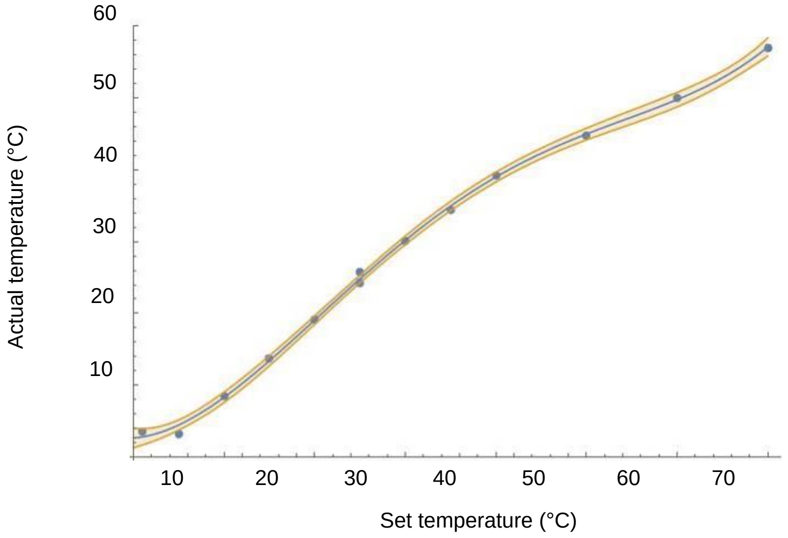

Fig. S21. Calibration curve of the temperature-controlled sample holder. The calibration curve was obtained using 2f-FCS. The water viscosity was determined at different temperatures by measuring the diffusion coefficient of Oregon Green. Viscosities were converted to temperature using the known temperature dependence of water viscosity. The solid line indicates a polynomic fit of fourth order. The collar band represents 90% confidence interval that is used for estimating the error in temperature.

References

[1] C. Löw, P. Moberg, E. M. Quistgaard, M. Hedrén, F. Guettou, J. Frauenfeld, L. Haneskog, P. Nordlund, *Biochim. Biophys. Acta - Gen. Subj.* **2013**, *1830*, 3497–3508.
[2] Y. Ural-Blimke, A. Flayhan, J. Strauss, V. Rantos, K. Bartels, R. Nielsen, E. Pardon, J. Steyaert, J. Kosinski, E. M. Quistgaard, C. Löw, *J. Am. Chem. Soc.* **2019**, *141*, 2404–2412.
[3] D. Weitz, D. Harder, F. Casagrande, D. Fotiadis, P. Obrdlik, B. Kelety, H. Daniel, *J. Biol. Chem.* **2007**, *282*, 2832–2839.
[4] A. Flayhan, H. D. T. Mertens, Y. Ural-Blimke, M. Martinez Molledo, D. I. Svergun, C. Löw, *Structure* **2018**, *26*, 345-355.e5.
[5] A. Qasim, I. Sher, O. Hirschhorn, H. Shaked, Z. Qasem, S. Ruthstein, J. H. Chill, *ChemBioChem* **2019**, *20*, 813–821.
[6] M. Rehahn, W. Mattice, U. Suter, *Rotational Isomeric State Models in Macromolecular Systems*, **1997**.
[7] V. B. Chen, W. B. Arendall, J. J. Headd, D. A. Keedy, R. M. Immormino, G. J. Kapral, L. W. Murray, J. S. Richardson, D. C. Richardson, *Acta Crystallogr. Sect. D Biol. Crystallogr.* **2010**, *66*, 12–21.
[8] S. Kalinin, T. Peulen, S. Sindbert, P. J. Rothwell, S. Berger, T. Restle, R. S. Goody, H. Gohlke, C. A. M. Seidel, *Nat. Methods* **2012**, *9*, 1218–1225.
[9] N. K. Lee, A. N. Kapanidis, Y. Wang, X. Michalet, J. Mukhopadhyay, R. H. Ebright, S. Weiss, *Biophys. J.* **2005**, *88*, 2939–2953.
[10] A. N. Kapanidis, T. A. Laurence, K. L. Nam, E. Margeat, X. Kong, S. Weiss, *Acc. Chem. Res.* **2005**, *38*, 523–533.
[11] A. Kapanidis, D. Majumdar, M. Heilemann, E. Nir, S. Weiss, *Cold Spring Harb. Protoc.* **2015**, *2015*, 979–987.
[12] D. Nettels, S. Müller-Späth, F. Küster, H. Hofmann, D. Haenni, S. Rüegger, L. Reymond, A. Hoffmann, J. Kubelka, B. Heinz, K. Gast, R. B. Best, B. Schuler, *Proc. Natl. Acad. Sci. U. S. A.* **2009**, *106*, 20740–20745.
[13] M. Aznauryan, L. Delgado, A. Soranno, D. Nettels, J. R. Huang, A. M. Labhardt, S. Grzesiek, B. Schuler, *Proc. Natl. Acad. Sci. U. S. A.* **2016**, *113*, 5389–5398.
[14] M. Aznauryan, D. Nettels, A. Holla, H. Hofmann, B. Schuler, *J. Am. Chem. Soc.* **2013**, *135*, 14040–14043.
[15] T. Dertinger, V. Pacheco, I. Von Der Hocht, R. Hartmann, I. Gregor, J. Enderlein, *ChemPhysChem* **2007**, *8*, 433–443.
[16] E. Rusinova, V. Tretyachenko-Ladokhina, O. E. Vele, D. F. Senear, J. B. Alexander Ross, *Anal. Biochem.* **2002**, *308*, 18–25.
[17] E. R. Likhachev, *Tech. Phys.* **2003**, *48*, 514–515.
[18] R. Vancraenenbroeck, H. Hofmann, *J. Phys. Chem. B* **2018**, *122*, 11460–11467.
[19] R. Vancraenenbroeck, Y. S. Harel, W. Zheng, H. Hofmann, *Proc. Natl. Acad. Sci. U. S. A.* **2019**, *116*, 19506–19512.
[20] B. Schuler, *Methods Mol. Biol.* **2007**, *350*, 115–138.
[21] B. K. Müller, E. Zaychikov, C. Bräuchle, D. C. Lamb, *Biophys. J.* **2005**, *89*, 3508–3522.
[22] S. Benke, D. Nettels, H. Hofmann, B. Schuler, *Nanotechnology* **2017**, *28*, 114002.
[23] I. V. Gopich, A. Szabo, *Proc. Natl. Acad. Sci. U. S. A.* **2012**, *109*, 7747–7752.
[24] S. Kalinin, A. Valeri, M. Antonik, S. Felekyan, C. A. M. Seidel, *J. Phys. Chem. B* **2010**, *114*, 7983–7995.
[25] A. Soranno, B. Buchli, D. Nettels, R. R. Cheng, S. Müller-Späth, S. H. Pfeil, A. Hoffmann, E. A. Lipman, D. E. Makarov, B. Schuler, *Proc. Natl. Acad. Sci. U. S. A.* **2012**, *109*, 17800–17806.
[26] B. Schuler, A. Soranno, H. Hofmann, D. Nettels, *Annu. Rev. Biophys.* **2016**, *45*, 207–231.
[27] E. Haas, M. Wilchek, E. Katchalski-Katzir, I. Z. Steinberg, *Proc. Natl. Acad. Sci. U. S. A.* **1975**, *72*, 1807–1811.
[28] A. Hoffmann, D. Nettels, J. Clark, A. Borgia, S. E. Radford, J. Clarke, B. Schuler, *Phys. Chem. Chem. Phys.* **2011**, *13*, 1857–1871.

Author Contributions

C.L. and H.H. designed the research and wrote the paper. T.L.M. and K.B. contributed equally to this work by designing research, performing experiments, analyzing data, and writing the paper. J.J. performed the rotational isomeric state model and rigid body simulations. F.W. built the temperature-controlled sample device for smFRET experiments. G.R. provided biochemical support.
